# Estimating photosynthetic characteristics of forage rape by fusing the sensitive spectral bands to combined stresses of nitrogen and salt

**DOI:** 10.3389/fpls.2025.1547832

**Published:** 2025-03-27

**Authors:** Jingang Wang, Haijiang Wang, Xin Lv, Jing Cui, Xiaoyan Shi, Jianghui Song, Weidi Li, Wenxu Zhang

**Affiliations:** ^1^ Agricultural College, Shihezi University, Shihezi, Xinjiang, China; ^2^ Key Laboratory of Oasis Ecological Agriculture of Xinjiang Production and Construction Corps, Shihezi University, Shihezi, Xinjiang, China

**Keywords:** hyperspectral technology, feature fusion, combined stresses, photosynthetic systems, continuous wavelet transform

## Abstract

Leaf gas exchange and chlorophyll fluorescence parameters (PGE-CFPs), which respond significantly and quickly to environmental stresses, have been used to assess the early responses of crop physiology to stresses. Most spectral estimations only focus on crop photosynthetic characteristics under a single environmental stress. Thus, the methods proposed previously are not suitable for the estimations under combined stresses (i.e., nitrogen and salt). In this research, the leaf spectral features of forage rape (*Brassica napus* L.) under nitrogen stress (NSpe) and salt stress (SSpe) were fused to increase the accuracy of the spectral estimation of photosynthetic characteristics of forage rape under combined stresses in arid region of Xinjiang, China. The results showed that PGE-CFPs’ spectral features were extracted with SPA (successive projections algorithm) after preprocessing. Among the SSpe- and NSpe-based models, the RF (random forest) models had higher estimation accuracy than the PLSR (partial least squares regression) and BPNN (backpropagation neural network) models. Specifically, the RF models had a PGE-CFPs estimation accuracy of 0.597–0.712, 0.640–0.715, and 0.377–0.461 under nitrogen stress (NS), salt stress (SS), and NS*SS, respectively. After fusing NSpe and SSpe, the accuracy in estimating PGE-CFPs of forage rape under NS, SS, and NS*SS were 0.729–0.755, 0.667–0.768, and 0.621–0.689, respectively. Then, the constructed models were further validated using field data, and the accuracy obtained was in the range of 0.585–0.711. Therefore, the feature fusion modeling method proposed has strong transferability and applicability. This research will offer a technical reference for crop photosynthesis monitoring at the early stage of environmental stresses.

## 1 Introduction

Lack of forage grass is a main factor limiting China’s animal husbandry development. Forage rape is a high-quality forage grass with high crude protein and fat contents and low crude fiber content, which has been planted in northern China to promote the animal husbandry development as well as protect the arid environment (Cotty and Dorin, 2012; Xing and Goldsmith, 2013; Li and Lin, 2014). However, soil salinization is widespread in northwest China, especially in Xinjiang province, posing challenges to forage rape planting (Konapala et al., 2020). Besides, in recent decades, excessive nitrogen (N) fertilization causes a great loss of N (N use efficiency is only 30%–35%), causing N pollution in Xinjiang (Xu et al., 2018). Therefore, at present, nitrogen stress (NS) and salt stress (SS) are widespread in Xinjiang, seriously impacting the planting of forage grasses, especially forage rape (Baker and Rosenqvist, 2004; Zhang et al., 2012).

Leaf gas exchange and chlorophyll fluorescence parameters (PGE-CFPs) are indicators of crop photosynthesis (Porcar-Castell et al., 2014). According to previous studies (Huang et al., 2004; Taras et al., 2010), SS destroys chloroplast ultrastructure and inhibits the photochemical efficiency of photosystem II (PS II), reducing crop photosynthetic rate. Nitrogen stress reduces the concentration of pigments in chloroplasts, causing stomatal limitation and photoinhibition of PS II. The PGE-CFPs respond to stresses earlier than other physiological indicators and morphological damage (Zahra et al., 2014). Especially, under NS or SS, PGE-CFPs changes of crops predate changes in salt ions and nitrogen concentrations. Therefore, PGE-CFPs can be used to evaluate crop photosynthetic performance at the early stage of stresses (Zarco-Tejada et al., 2000).

Early, non-destructive, and accurate assessment of crop responses especially photosynthetic performance to external stresses is of great significance for agricultural production. Traditionally, PGE-CFPs are non-destructively measured using portable fluorescence instruments. However, this method requires shading and other processing, which is complex, time-consuming, and difficult to realize rapid large-scale monitoring (Li, 2021). Remote sensing allows quick and accurate crop growth monitoring (Tirado et al., 2020). Under stress conditions, crop physiological activities, especially photosynthesis, obviously change, inducing the responses of leaf spectral reflectance. This provides the direct basis for spectral estimation. For instance, Zheng et al. (2021) found that the first-order derivative-based spectral vegetation index D690/D1320 could accurately estimate the chlorophyll fluorescence parameter Fv/Fm of salt-stressed *Suaeda salsa* leaves. Feng et al. (2015) reported that the chlorophyll fluorescence index NDF12/4 constructed by hyperspectral technique could be used for diagnosing N status in wheat. Besides, remote sensing techniques have also been applied in the assessment of photosynthetic characteristics of barley, wheat, maize, and cotton (Peñuelas et al., 1997; Zhu et al., 2007; Tan et al., 2012; Xue et al., 2013).

Most spectral estimations focus on crop photosynthetic characteristics under a single stress. However, crops often face multiple stresses simultaneously, resulting in low applicability of the estimation models constructed based on single stress conditions (Zandalinas and Mittler, 2022; Hu et al., 2023). Therefore, it is very urgent to explore the spectral responses of PGE-CFPs of crop leaves under combined stresses, to further improve the accuracy of spectral estimation of crop photosynthetic characteristics. This study hypothesized that fusing the spectral features of PGE-CFPs of forage rape leaves under NS and SS might improve the estimation accuracy of photosynthetic performance at the early stage of combined stresses. The specific objectives were to explore: (1) The photosynthetic response of forage rape to NS, SS, and NS*SS; (2) The spectral features of PGE-CFPs of forage rape leaves under the three types of stress; And (3) the impacts of various modeling strategies on the accuracy of spectral estimation of forage rape photosynthetic characteristics under various stresses. This research will offer a technical reference for improving the estimation accuracy of forage rape photosynthesis performance under combined stresses.

## 2 Materials and methods

### 2.1 Materials and research site

Forage rape variety Huayouza 62 with low erucic acid and glucoside content, strong cold and drought resistance, and short growth cycle was used in this study.

**Figure 1:**
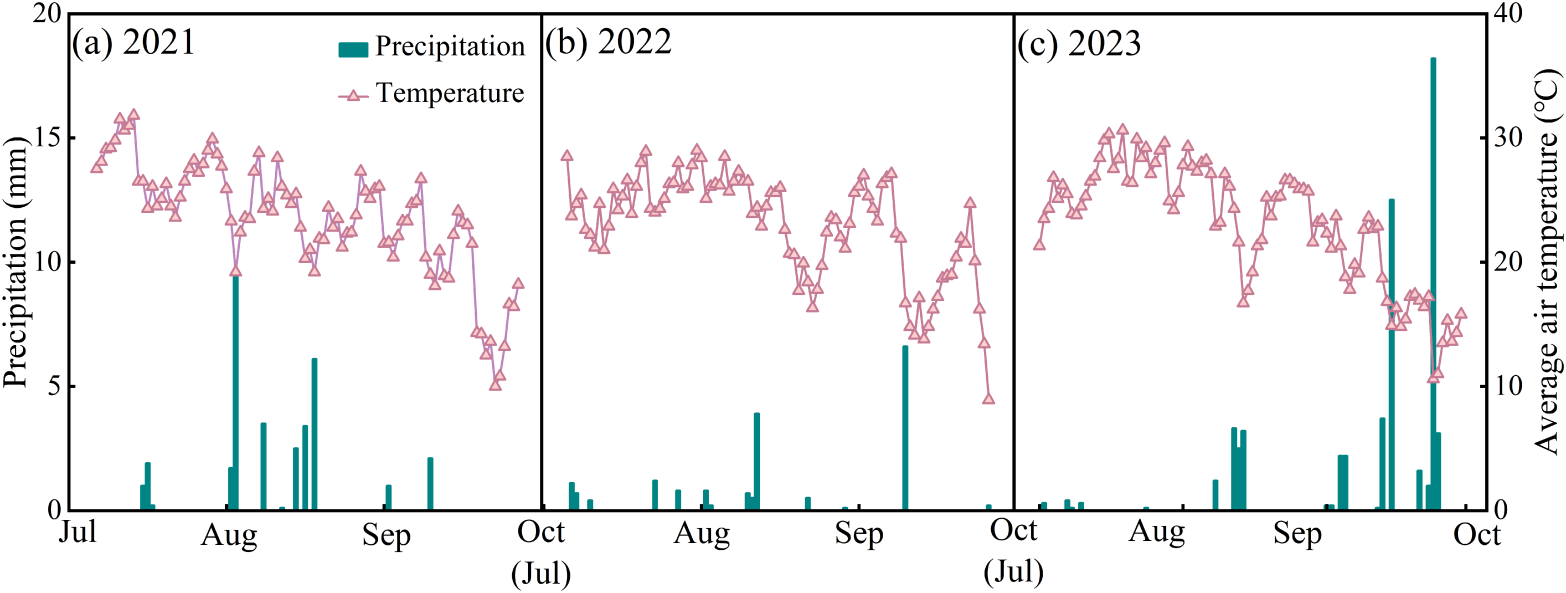
Daily average air temperature and precipitation during forage rape growing season from 2021 to 2023.

The experiment was conducted from 2021 to 2023 at Shihezi University Experimental Station in Xinjiang, China (86°3′ N, 44°18′ E, a.s.l. 428 m) in a temperate continental climate zone. Meteorological data were obtained from the Ulaanwusu Meteorological Station near the study area (**Figure 1**). The annual average sunshine hours was 2725 – 2820 h, the annual average temperature was 6.5 – 7.2°C, the annual average precipitation was 125 – 208 mm, and the annual average evaporation was 1200 – 1500 mm. The average temperatures of the whole growing season of forage rape in 2021, 2022, and 2023 were 23.6, 23.0, and 23.1 °C, respectively, and the total precipitations during the growing season were 32.9, 17.7, and 56.4 mm, respectively. The physicochemical properties of the experimental-site soil (soil type: gray desert soil) were as follows: The pH was 7.64, the organic matter content was 12.05 g·kg^–1^, the total nitrogen content was 0.89 g·kg^–1^, the available nitrogen content was 93.6 mg·kg^–1^, the available phosphorus content was 18.7 mg·kg^–1^, and the available potassium content was 242 mg·kg^–1^. Strong evaporation leads to a large accumulation of soluble salt in the soil of the region, severely limiting the growth of crops.

### 2.2 Experimental design

In this experiment, a two-factor (soil salt, N application rate) randomized complete block design was adopted. For the soil salt factor, there were two levels (S1 and S2). The soil of S1 was sampled from the local farmlands, with salt type of sulfate chloride and salt content of 2.1 g·kg^–1^ (Liu et al., 2024). The soil of S2 was the mixture of the saline soil collected from surrounding areas (salt type: sulfate chloride; salt content (0-30 cm soil layer): 22.4 g·kg^-1^) and the S1 soil. Based on the Classification Criterion for Saline Soils in Xinjiang (non-salinized soil (0–3 g·kg^-1^), mildly salinized soil (3–5 g·kg^-1^), moderately salinized soil (5–10 g·kg^-1^), and severely salinized soil (10–20 g·kg^-1^)) (Luo, 1985), the salt of the S2 soil was made to 8.75 g·kg^-1^ (measured by conductivity method) (Luo, 1985; Liu et al., 2024). For the N application rate factor, there were three levels: 240 kg·ha^-1^ (N1.2), 200 kg·ha^-1^ (Nc, N application rate commonly adopted by local farmers), and 160 kg·ha^-1^ (N0.8). The organic form of N, urea (N, 46%), was applied. There were a total of six treatments, and three replications/plots were made for each treatment. Each plot (2.0 m × 3.0 m) was surrounded by brick walls to isolated it from the adjacent plots to avoid interference. The brick walls were 1.2 m high, of which 0.2 m was above the ground. There was a cement mortar layer on the brick wall surface. Impermeable membranes (1 mm thick) were covered on the surface of brick walls below the ground. Soils were backfilled in the plots in layers (20 cm per layer), and the bulk density was consistent with that of the farmland of the Experimental Station.

To clearly clarify the impacts of different stress types on the photosynthetic characteristics of forage rape, the six treatments were classified into four groups: Control (CK) group, including S1Nc treatment; Nitrogen stress (NS) group, including S1N0.8 and S1N1.2 treatments; Salt stress (SS) group, including S2Nc treatment; Combined stresses (NS*SS) group, including S2N0.8 and S2N1.2 treatments.

The phosphorus and potassium fertilizer application rates recommended by Zhu et al. (2019) were adopted, i.e., 90 kg·ha^–1^ of triple superphosphate (P_2_O_5_, 46%–54%), and 75 kg·ha^–1^ of potassium sulfate (K_2_O, 50%). All P and K fertilizers were applied before sowing. About 40% of nitrogen fertilizer (N, 46%) were applied before sowing, and the remaining 60% were topdressed through drip fertigation. During the growing season, plants were irrigated six times in total, and the total amount was 4500 m^3^·ha^-1^, keeping the field capacity at 70% – 80%. The seeding dates in 2021, 2022, and 2023 were July 13, July 17, and July 14, respectively (**Table 1**).

**Table 1:** Nitrogen fertilizer application rate and salt content for different treatments and stress types.

Type of stress	Treatment	Salt content (g·kg-1)	N fertilizer application rate (kg·ha-1)
CK (Non stress condition)	S1Nc	2.1	200
NS	S1N0.8	2.1	160
NS	S1N1.2	2.1	240
SS	S2Nc	8.75	200
NS*SS	S2N0.8	8.75	160
NS*SS	S2N1.2	8.75	240

CK, NS, SS, and NS*SS represent control, nitrogen stress, salt stress, and nitrogen-salt combined stresses groups, respectively; S1, Non-salt stress condition; S2, Salt stress; Nc, Recommended nitrogen application rate; N0.8, Low-nitrogen stress; N1.2, High-nitrogen stress.

### 2.3 Data acquisition

#### 2.3.1 Spectral data acquisition

The hyperspectral data of forage rape leaves was collected with a portable spectrometer (PSR–3500, Spectral Evolution, USA) (wavelength range: 350 – 2500 nm). This spectrometer had following detectors: (i) 512 silicon photodiode array, with a spectral range of 350 nm – 1000, a resolution of 3.5 nm, and an interval of 1.5 nm; (ii) 256-element InGaAs array, with a spectral range of 970 nm – 1910, a resolution of 7 nm, and an interval of 3.8 nm; and (iii) 256 element InGaAs array, with a spectral range of 1900 nm – 2500 nm, a resolution of 10 nm, and an interval of 2.5 nm. After resampling the spectrum to 1 nm, a total of 2151 bands were obtained in the region of 350 – 2500 nm. The spectrometer was calibrated every ten samples. On a cloudless day, the clamp connected to the spectrometer was used to determine different parts (left, middle, and right) of forage rape leaves at 12:00 – 14:00, and spectral reflectance was obtained after averaging. Spectral data were acquired on the 10th, 20th, 30th, and 40th day after sowing (20 spectral data for each stress type at each time). Finally, 240 spectral data were obtained for each type of stress (80 samples per year), with a total of 960 samples obtained.

#### 2.3.2 Determination of PGEs

The leaf PGEs (Pn (net photosynthetic rate), Ci (intercellular carbon dioxide concentration), gs (stomatal conductance), and Tr (transpiration rate)) of the forage rape plants subjected to spectral data acquisition were determined by a Li-6400 gas exchange detector at 9:00 – 11:00. The photosynthetically active radiation of the instrument was 1200 μmoL·m^–2^·s^–1^, the CO_2_ concentration was 400 μmoL·moL^–1^, the chamber temperature was 30°C, the air flow rate was 500 μmoL·s^–1^, and the relative humidity was 55%.

#### 2.3.3 Determination of CFPs

The leaf CFPs of forage rape plants subjected to spectral data acquisition were determined with a Chl fluorometer (PAM-2500, Walz, Germany) and a 2030-B leaf clip. Firstly, under photoreaction, the steady-state chlorophyll fluorescence (Fs) was determined. After that, a light of 1200 μmoL·m^-2^·s^-1^ was emitted, with a pulse time of 0.8 s, to measure the maximum (Fm′) and minimum (F0′) fluorescence yield in light-adapted state. After 30-min dark adaption, a strong light was emitted to measure the maximum (Fm) and minimal (F0) fluorescence. Then, the photochemical quenching coefficient (qP), non-photochemical quenching (NPQ), effective quantum yield of PS II photochemistry (ΦPS II), maximum photochemical efficiency of PS II (Fv/Fm), PS II potential activity (Fv/F0), and electron transport rate (ETR) were computed (Wang et al., 2023).

#### 2.3.4 Statistical analysis of PGEs-CFPs data

**Figure 2:**
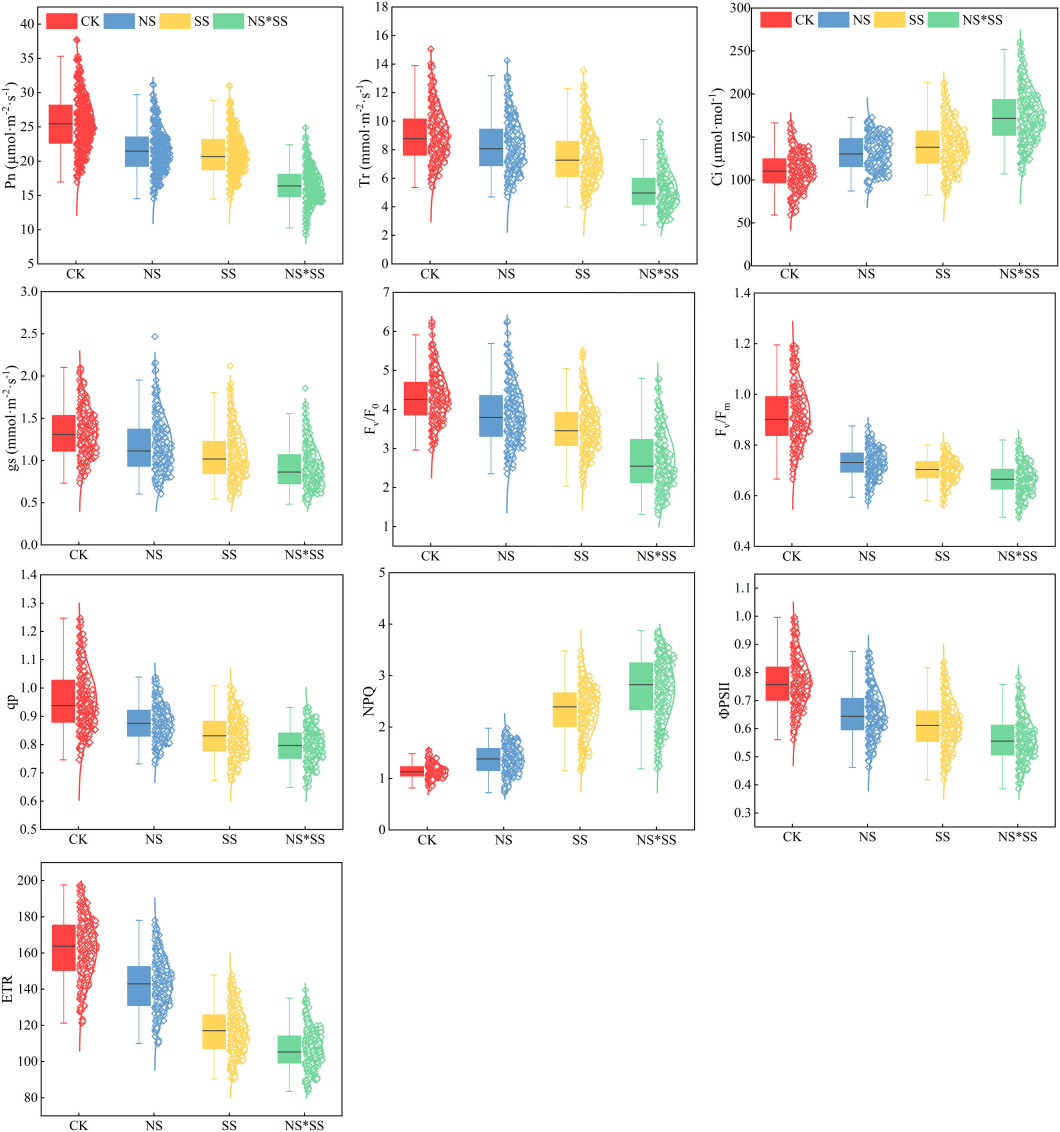
Statistical analysis of gas exchange and chlorophyll fluorescence parameters (PGE-CFPs) of forage rape leaves (n = 240). The central lines in the boxplot represent the medians, and the upper and lower boundaries of the extension lines represent the maximum and minimum values, respectively. CK, NS, SS, and NS*SS are control, nitrogen stress, salt stress, and nitrogen-salt combined stresses, respectively; Pn, Net photosynthetic rate; Ci, Intercellular carbon dioxide concentration; gs, Stomatal conductance; Tr, Transpiration rate; ΦPS II, Effective quantum yield of PS II photochemistry; Fv/Fm, Maximum photochemical efficiency of PS II; Fv/F0, PSII potential activity; qP, Photochemical quenching coefficient; NPQ, Non-photochemical quenching; ETR, Electron transport rate.

The PGE-CFPs data (**Figure 2**) were partitioned into three ranges according to the values from high to low, and the values with a large error were removed. After that, the remaining data were split into two sets, calibration and verification set (2: 1). It can be seen from **Figure 2** that the sample dispersion degree of each parameter is high. This indicates that the sample selection meets the requirements of sufficient quantity, wide range, and uniform distribution.

This study aimed to explore the spectral response mechanisms of photosynthetic parameters of forage rape leaves to different types of stress, thus only the sample data of stress groups (NS, SS, NS*SS) were selected to construct the estimation models. The CK group data was used for comparison in the analysis of stress effects, and its spectral and physiological parameter data were not included in the following modelings to avoid interference with the extraction of spectral features of stress conditions by noises of non-stress conditions (Okyere et al., 2024).

### 2.4 Spectral preprocessing

It is easy to generate a large amount of noises during spectral data acquisition, due to the influences of environmental and instrumental factors. To remove noises and highlight useful information, five preprocessing methods including SG smoothing, SG-SNV transform, SG-MSC, SG-peak area normalization (PAN), and SG-CWT were employed to preprocess raw spectral reflectance.

#### 2.4.1 Savitzky-Golay

Polynomial least squares fitting was conducted on the spectral data in the moving window through polynomials, to achieve the purpose of smoothing. In SG smoothing, the polynomial degree and smoothing window width have a decisive influence on the smoothing performance (Barnes et al., 1993). In this study, after many attempts, the polynomial degree and smoothing window width were selected to be 4 and 6, respectively. The smoothed bands were calculated by following formula (Equation 1):


(1) $$x_{asmth}={\bar{x}}_{a}=\frac{1}{H}\sum_{i=-w}^{+w}x_{a+i}h_{i}$$

where $\bar{x\;}$ *_a_* is the average of the spectrum at central wavelength *a* and the spectrum at the wavelengths with a distance of *w* before and after *a*, *hi* is the coefficient obtained by least square fitting, and *H* is the normalization factor.

#### 2.4.2 Standard normal variate

Standard normal variate processes each spectrum to reduce errors induced by optical path changes, surface scattering, etc. It standardizes raw spectra, subtracts the mean of entire spectrum to eliminate shifts, and divides by the standard deviation, to realize similar proportions (Equation 2).


(2) $$x_{SNV}=\frac{x-\bar{x}}{\sqrt{\frac{\sum_{i=1}^{p}(x_{i}-\bar{x})}{p-1}}}$$

where *x* is raw spectrum, $\bar{x\;}$ is the mean of a sample’s all bands, and *i* is the number of bands (*i* = 1, 2,…, *p*).

#### 2.4.3 Multivariate scattering correction

Differences in particle size always impact the scattering of near-infrared light, leading to spectral differences. The MSC can eliminate the particle size-induced influence and preserve information related to physiological indices in the raw spectrum as much as possible. It assumes that scattering is independent of wavelength and sample concentration. Firstly, the average spectrum $\bar{X}$ of the samples of correction set was calculated, and then the linear regression of the $\bar{X}$ and the spectrum of sample *x*(1×m) was conducted, that is, $x=a\bar{X}+\beta$ . Finally, *α* and *β* were calculated (Equation 3).


(3) $$x_{MSC}=\frac{x-\beta }{a}$$

By adjusting the value of *α* and *β*, it is possible to reduce spectral variability while preserving as much information about physiological indices as possible in the raw spectrum.

#### 2.4.4 Peak area normalization

Peak area normalization is the modification of spectrum when the path length cannot be determined, or the separation of spectral features of a physiological index to smooth spectrum through computing the area below a sample’s spectral curve.

#### 2.4.5 Continuous wavelet transform

Continuous wavelet transform decomposes signals into multi-scale wavelets. It highlights weak signals and regional features (Koger et al., 2003). In this study, the wavelet basis function was employed to decompose the hyperspectral data, and wavelet coefficients of different scales were generated. The wavelet coefficients were two-dimensional data (band (*j* = 1, 2,…, n), scale (*i* = 1, 2,…, m)) (Equations 4, 5).


(4) $$Wf(a,b)=\int_{-\infty }^{+\infty }f(\lambda )\Psi_{a,b}(\lambda )d\lambda$$

(5) $$\Psi_{\left(a,b\right)}(\lambda )=\frac{1}{\sqrt{a}}\Psi (\frac{\lambda -b}{a})$$

where *Wf (a, b)* is wavelet coefficient, *f(λ)* is reflectance, *λ* is 350 – 2500 nm, *Ψ_a,b_(λ)* is wavelet basis function converted with *a* (scale factor) and *b* (expansion factor). The Gaus1 wavelet function was used in this research. The decomposition scales were 2^1^, 2^2^, …, 2^10^, i.e., scale 1, 2,…, 10.

### 2.5 Selection of spectral features by successive projections algorithm

Successive projections algorithm is a forward variable extraction approach. It eliminates redundancy using vector projection and extracts spectral features. It can decrease the spectral band number for modeling, and minimize collinearity among spectral features, increasing efficiency and accuracy. In this research, when the SPA-extracted band number was 5 – 30, the RMSE was the smallest. Specific algorithms are described in Galvo et al. (2007).

### 2.6 Modeling and evaluation

#### 2.6.1 Partial least squares regression

Partial least squares regression could address the multicollinearity and small-sample-size issues, and has an obvious advantage in dealing with data with multiple dependent variables. It can simplify data structure, and reduce data dimension and noises. The core of PLSR is to model the relationship between the independent variables (X) and dependent variables (Y) by extracting latent variables (LVs), and to select the model with the minimum PRESS value and the least LVs (avoid overfitting) through leave-one-out cross-validation (LOOCV) (Inoue et al., 2016) (Equations 6, 7).


(6) $$Y_{i}=\beta_{0}+\sum_{k=1}^{r}\beta_{k}T_{ik}+e_{i}(i=1,\ldots ,n)$$

(7) $$T_{ik}=\sum_{j=1}^{m}C_{kj}X_{ij}(k=1,\ldots ,r)$$

where *Y_i_* and *X_ij_* denote dependent and independent variable, respectively, *m* denotes wavelength, *n* denotes the number of samples of PGE-CFPs, *r* denotes the latent variable (LV) number, *β_k_*, *T_ik_*, *C_kj_*, and *e_i_* denote regression coefficient, latent variable, coefficient of LV, and error, respectively.

#### 2.6.2 Random forest

Random forest is a machine learning algorithm. This modeling method has a fast training speed and does not require cross-validation. Besides, the randomness of sampling and feature selection make it difficult to fall into overfitting (Breiman, 2001). In this research, the RF modeling procedure was as follows: Firstly, bootstrap resampling was used to extract multiple samples from the sample set. Then, multiple decision trees for estimation were constructed based on the decision tree for each bootstrap sample. Finally, the classification result was obtained by majority voting. The core hyperparameters of the model were the number of decision trees (n_estimators), maximum tree depth (max_depth), and maximum number of features per tree (max_features), with initial ranges of [100, 200, 300], [5, 10, 15], and [√p, p/3] (where p is the number of features), respectively. The optimal values, i.e., n_estimators = 200, max_depth = 10, and max_features = √p, were determined through repeated training.

#### 2.6.3 Backpropagation neural network

Backpropagation neural network is a multiple layer network that can minimize the mean square error of the actual and expected outputs with the gradient search technique. The BPNN computation comprises forward and inverse computations. The forward propagation processes the input, hidden, and output layers sequentially. The state of a layer’s neurons only affects that of the next layer. When desired output could not be generated, reverse propagation is initialized. That is, the erroneous signals are returned, and minimized by altering neurons’ weights (Wang et al., 2015). In this research, the key hyperparameters included the number of hidden-layer nodes, learning rate, and maximum number of epochs (Epochs). The structure was tested using a trial-and-error method, evaluating single-layer (5–15 nodes) and double-layer (5–10–5 nodes) configurations. The learning rate was searched on a logarithmic scale from 0.001 to 0.1. Early stopping was applied, i.e., terminating training if the validation set loss did not decrease for 5 consecutive iterations. Based on the tests, the optimal configuration was determined, i.e., a hidden layer with 10 nodes, learning rate = 0.01, and Epochs = 500.

#### 2.6.4 Modeling based on feature fusion

**Figure 3:**
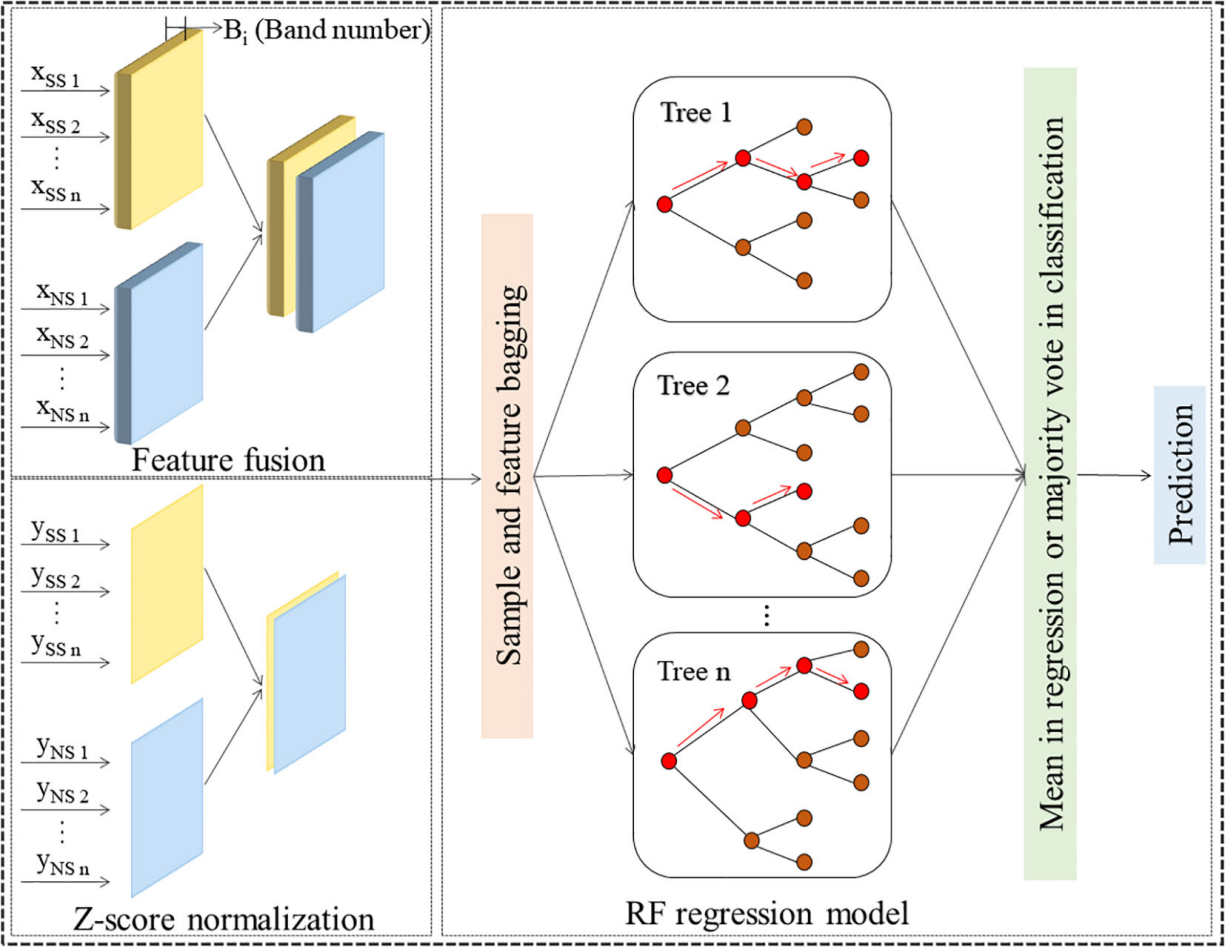
Construction of models by fusing the spectral features of gas exchange and chlorophyll fluorescence parameters (PGE-CFPs) of forage rape leaves under nitrogen and salt stresses.


To improve the estimation accuracy of forage rape PGE-CFPs in the early stage of combined stresses, feature fusion was proposed for modeling. Firstly, considering the differences in PGE-CFPs under NS and SS, this study normalized the data of these parameters under NS and SS, and then took the mean value *y_i_* of the two as the input of model *Y* (Equation 8). The spectral features of PGE-CFPs under NS and SS extracted by SPA were connected in series and used as the inputs of independent variable *X* for modeling (**Figure 3**). This research adopted standard z-score normalization for normalization, and standardized the data by mean $\bar{y}$ and standard deviation *S* of the raw data (Equation 9). The data after processing conformed to the normal distribution (Zeng and Tao, 2023).

(8) $$y_{i}=\frac{\frac{y_{ssi}-{\bar{y}}_{SS}}{S_{SS}}+\frac{y_{Ns}-{\bar{y}}_{NSi}}{S_{NS}}}{2}$$

(9) $$S=\sqrt{\frac{1}{N-1}\sum_{i=1}^{N}{\left(y_{i}-\bar{y}\right)}^{2}}$$

where $y_{i}$ is the input (dependent variable), $y_{ssi}$ , ${\bar{y}}_{SS}$ , and $S_{SS}$ are sample value, mean, and SD of PGE-CFPs under salt stress, respectively, and $y_{Nsi}$ , ${\bar{y}}_{NS}$ , and $S_{NS}$ are sample value, mean, and SD of PGE-CFPs under N stress, respectively.

#### 2.6.5 Model validation

To further validate the transferability and practical applicability of the constructed feature fusion model, rapeseed sample data was collected from local conventional farmlands in 2022 and 2023, and 160 samples were randomly selected for independent testing. To ensure the consistency of solar light during data acquisition, the planting dates in the pot experiment and the conventional farmland experiment were consistent. The PLSR, RF, and BPNN models were assessed using R^2^ (Coefficient of determination) (Equations 10, 11) and RMSE (Root mean squared error) (Equations 12, 13). The model with a larger R^2^ and a smaller RMSE generally had a higher accuracy (Chen et al., 2016).


(10) $$R_{c}^{2}=1-\frac{\sum_{i=1}^{n_{c}}{\left(y_{ci}-{\hat{y}}_{ci}\right)}^{2}}{\sum_{i=1}^{n_{c}}{\left(y_{ci}-{\bar{y}}_{c}\right)}^{2}}$$

(11) $$R_{p}^{2}=1-\frac{\sum_{i=1}^{n_{p}}{\left(y_{pi}-{\hat{y}}_{pi}\right)}^{2}}{\sum_{i=1}^{n_{p}}{\left(y_{pi}-{\bar{y}}_{p}\right)}^{2}}$$

(12) $$RMSE_{c}=\sqrt{\frac{1}{n_{c}}\sum_{i=1}^{n_{c}}{\left(y_{ci}-{\hat{y}}_{ci}\right)}^{2}}$$

(13) $$RMSE_{p}=\sqrt{\frac{1}{n_{p}}\sum_{i=1}^{n_{p}}{\left(y_{pi}-{\hat{y}}_{pi}\right)}^{2}}$$

Where *RMSE_c_* and *RMSE_p_* are the RMSE of the calibration set and the verification set, respectively, *R_c_^2^* and *R_p_^2^* are the R^2^ of the calibration set and the verification set, respectively, ${\hat{y}}_{ci}$ and ${\hat{y}}_{pi}$ are the prediction of the i_th_ sample of the calibration set and the verification set, respectively, $n_{c}$ and $n_{p}$ are the sample count of the calibration set and the verification set, respectively, ${\bar{y}}_{c}$ and ${\bar{y}}_{p}$ are the mean of measured values of the calibration set and the verification set, respectively, $y_{pi}$ is the measured value of the i_th_ sample in the verification set, $S_{D}$ is the standard deviation of the measured values of the verification set, and $RMSE_{CV}$ is the RMSE for cross validation.

### 2.7 Data analysis

One-way ANOVA was performed in SPSS 21.0 at *p* < 0.05 according to Clarke and Green (1988). The CWT, SPA, as well as feature fusion were completed in Matlab 2016a. Modeling was completed in Unscramber X 10.1. Graphics were made in Origin 2018.

## 3 Results

### 3.1 Effects of NS, SS, and NS*SS on the PGEs of forage rape leaves

**Figure 4:**
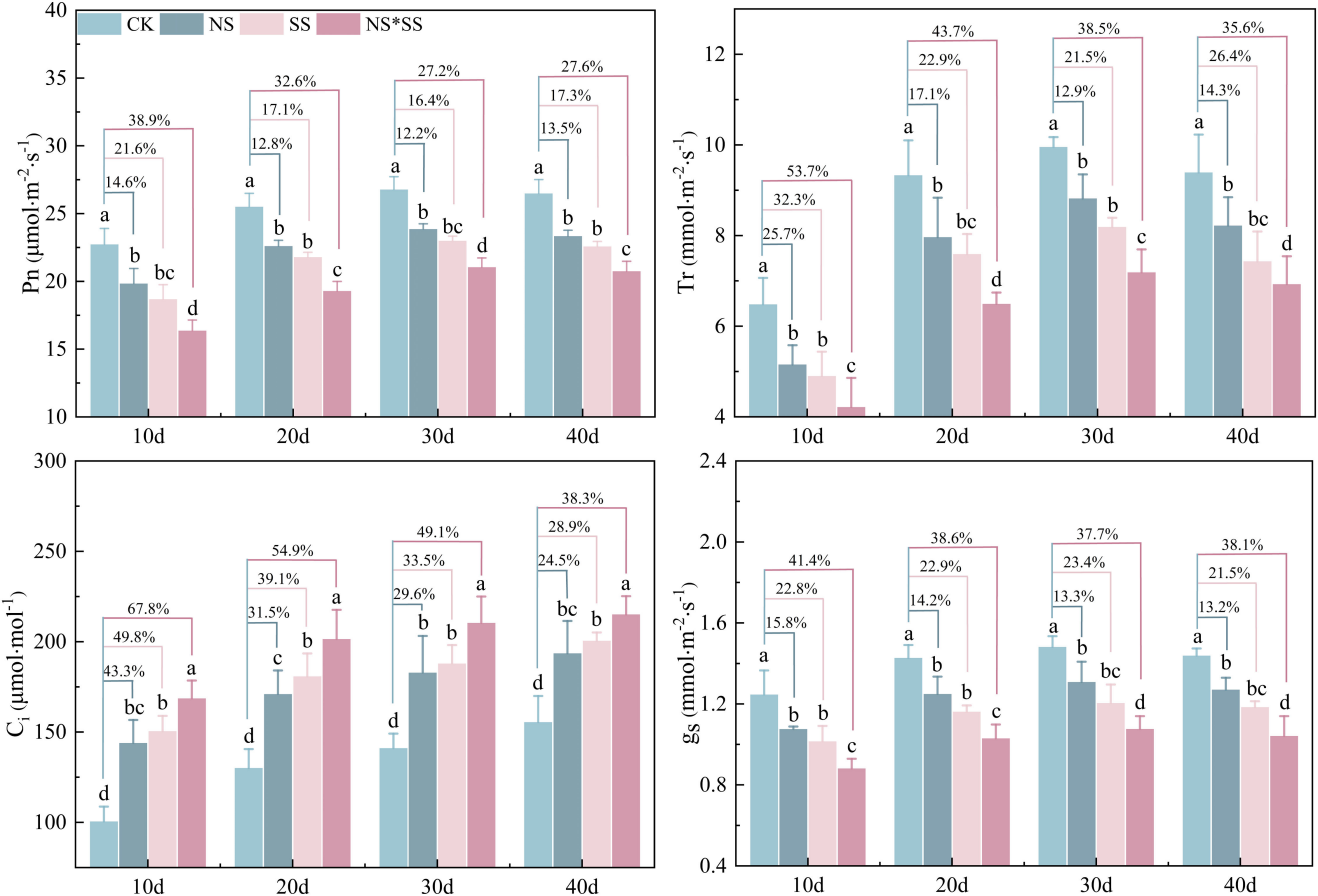
Changes in gas exchange parameters (PGEs) of forage rape leaves under nitrogen stress, salt stress, and nitrogen-salt combined stresses. Different lowercase letters indicate significant differences between treatments (*p* < 0.05), and the percentages in the same group indicate the changing amplitude of PGEs of forage rape leaves under NS, SS, and NS*SS compared with those of CK. The same below. CK, NS, SS, and NS*SS are control, nitrogen stress, salt stress, and nitrogen-salt combined stresses, respectively; Pn, Net photosynthetic rate; Ci, Intercellular carbon dioxide concentration; gs, Stomatal conductance; Tr, Transpiration rate; 10 d, 20 d, 30 d, and 40 d represent 10, 20, 30, and 40 days after sowing, respectively.

The NS, SS, and NS*SS all affected the PGEs of forage rape leaves (NS*SS > SS > NS). The leaf gs, Tr, and Pn of NS, SS, and NS*SS groups reduced (*p* < 0.05), while the Ci increased (*p* < 0.05), compared with those of CK group. The largest variation was found on day 10. Specifically, the Pn of NS, SS and NS*SS groups reduced by 14.6%, 21.6%, and 38.9%, the Tr reduced by 25.7%, 32.3%, and 53.7%, the gs reduced by 15.8%, 22.8%, and 41.4%, and the Ci increased by 43.3%, 49.8%, and 67.8%, respectively, compared with those of CK group (*p* < 0.05). Under NS, SS, and NS*SS conditions, the Pn, Tr, and gs of forage rape leaves first increased and then decreased, while Ci gradually increased (**Figure 4**).

### 3.2 Effects of NS, SS, and NS*SS on the CFPs of forage rape leaves

**Figure 5:**
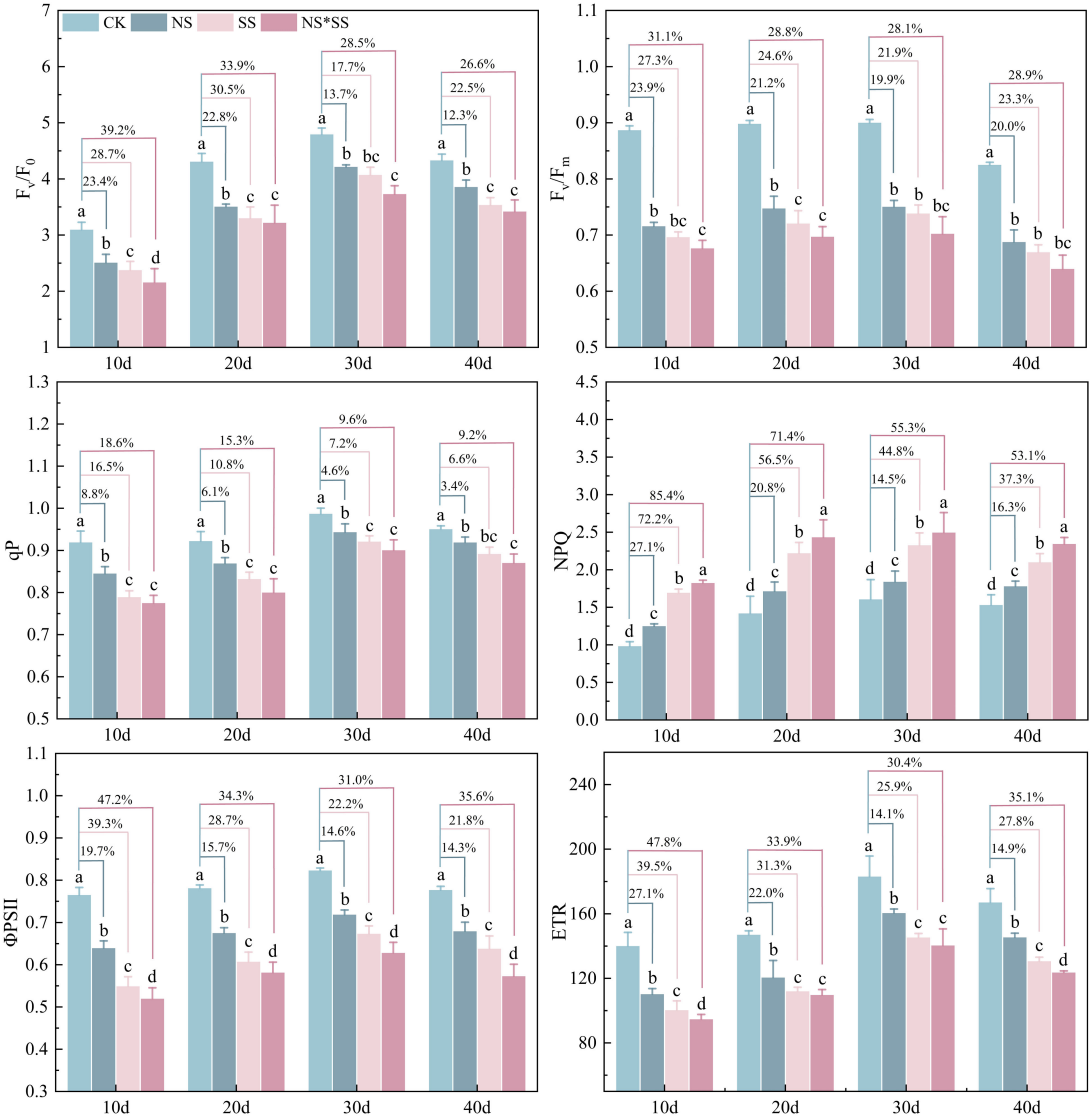
Changes of chlorophyll fluorescence parameters (CFPs) of forage rape leaves under nitrogen stress, salt stress, and nitrogen-salt combined stresses. CK, NS, SS, and NS*SS are control, nitrogen stress, salt stress, and nitrogen-salt combined stresses, respectively; Fv/F0, PS II potential activity; Fv/Fm, Maximum photochemical efficiency of PS II; qP, Photochemical quenching coefficient; NPQ, Non-photochemical quenching; ΦPS II, Effective quantum yield of PS II photochemistry; ETR, Electron transport rate. 10 d, 20 d, 30 d, and 40 d represent 10, 20, 30, and 40 days after sowing, respectively.

The NS, SS, and NS*SS all affected the CFPs of forage rape leaves (NS*SS > SS > NS) (**Figure 5**). The ETR, ΦPS II, qP, Fv/Fm, and Fv/F0 of forage rape leaves of NS, SS, and NS*SS groups reduced (*p* < 0.05), while the NPQ increased (*p* < 0.05), compared with those of CK group. The largest variation was found on day 10. Specifically, the Fv/F0 of forage rape leaves of the NS, SS and NS*SS groups reduced by 23.4%, 28.7%, and 39.2%, the Fv/Fm reduced by 23.9%, 27.3%, and 31.1%, the qP reduced by 8.8%, 16.5%, and 18.6%, the ΦPS II reduced by 19.7%, 39.3%, and 47.2%, the ETR reduced by 27.1%, 39.5%, and 47.8%, and the NPQ increased by 27.1%, 72.2%, and 85.4%, respectively, compared with those of CK group (*p* < 0.05). Under NS, SS, and NS*SS conditions, the CFPs of forage rape leaves first increased and then reduced (peaking on day 30).

### 3.3 Effects of NS, SS, and NS*SS on spectral reflectance of forage rape leaves

**Figure 6:**
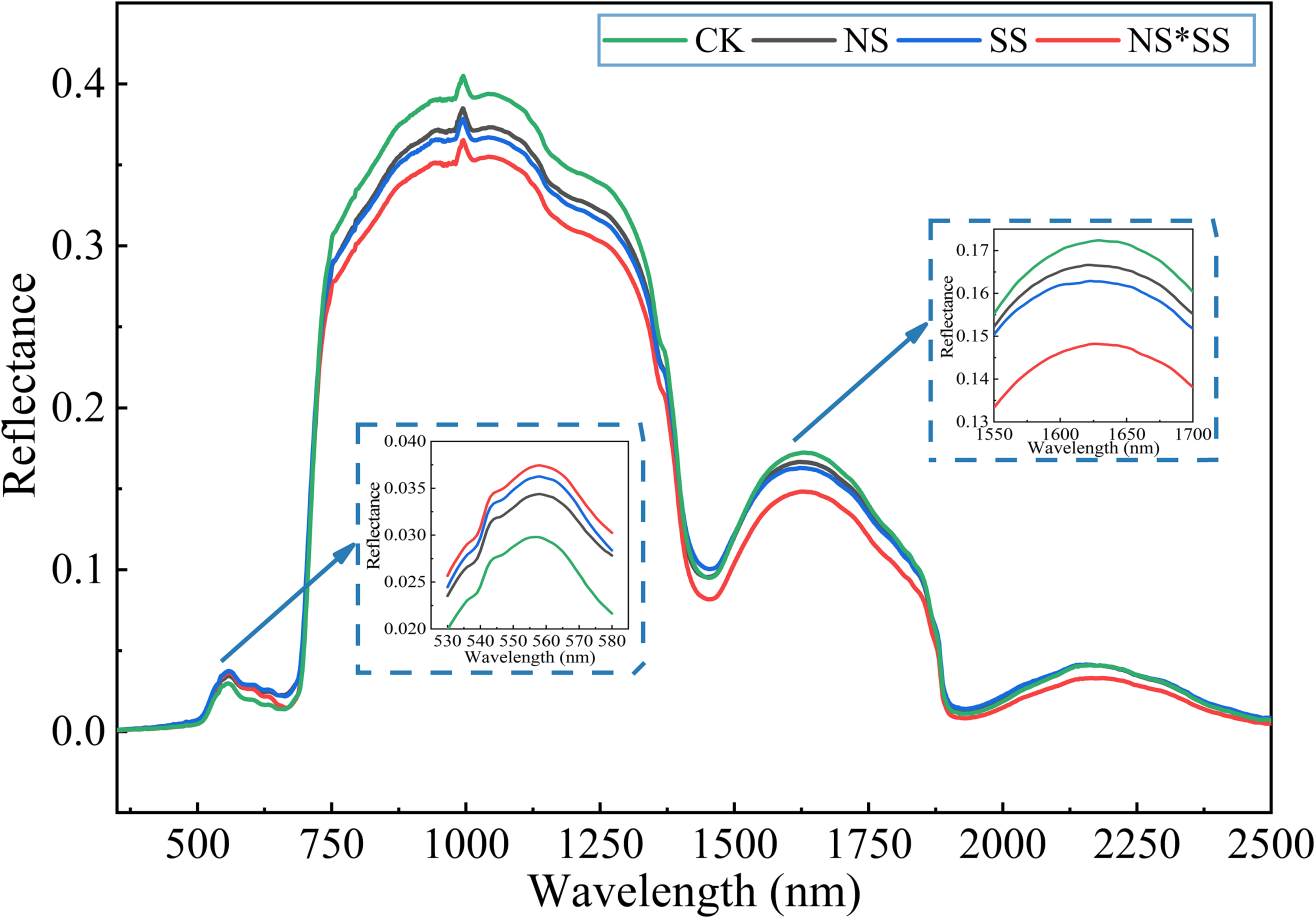
Changes of spectral reflectance of forage rape leaves under nitrogen stress, salt stress, and nitrogen-salt combined stresses. CK, NS, SS, and NS*SS are control, nitrogen stress, salt stress, and nitrogen-salt combined stresses, respectively.

The NS, SS, and NS*SS had a similar impact on the spectral reflectance of forage rape leaves, but the spectral reflectance were inconsistent (**Figure 6**). A reflectance peak was found in 400 – 700 nm (visible region), and the reflectance of NS, SS, and NS*SS groups decreased compared with that of CK group. Specifically, the reflectance at 558 nm of NS, SS, and NS*SS groups decreased by 15.48%, 21.79%, and 25.76%, compared with that of CK group. The reflectance increased sharply in 700 – 900 nm. The trend of spectral reflectance curve in 1100 – 2500 nm (peaking at 1626 nm) was opposite to that in the visible region. Besides, the reflectance at 1626 nm of NS, SS, and NS*SS groups increased by 3.46%, 5.83%, and 16.26%, respectively, compared with that of CK group.

### 3.4 Comparison of spectral preprocessing methods based on PLSR model

The full band was used to construct the PLSR models under NS, SS, and NS*SS, to determine the optimal spectral preprocessing method (**Table 2**). Different preprocessing methods all increased estimation accuracy. Although the optimal preprocessing method for different parameters varied under NS, SS, and NS*SS, the optimal preprocessing methods were CWT 4–6. Specifically, under NS, the optimal preprocessing method for ΦPS II and Fv/Fm was CWT6, and that for other parameters was CWT5. Under SS, the optimal preprocessing method for Pn, ΦPS II, and ETR was CWT6, and that for other parameters was CWT5. Under NS*SS, the optimal preprocessing method for NPQ was CWT5, and that for other parameters was CWT4. Due to space limitations, four parameters were selected for each type of stress for follow-up research, based on the sensitivity of PGE-CFPs to NS, SS, and NS*SS in Sections 3.1 and 3.2 and the estimation accuracy of the models in this section. The CWT5–Ci, CWT6–Fv/Fm, CWT5–NPQ, and CWT5–ETR, with a R^2^ of 0.702, 0.670, 0.641, and 0.655, respectively, were selected for the analysis of NS condition. The CWT5–Ci, CWT5–Fv/Fm, CWT5–NPQ, and CWT6–ETR, with a R^2^ of 0.665, 0.612, 0.629, and 0.639, respectively, were selected for the analysis of SS condition. The CWT4–Ci, CWT4–Fv/Fm, CWT5–NPQ, and CWT4–ETR, with a R^2^ of 0.589, 0.597, 0.254, and 0.511, respectively, were selected for the analysis of NS*SS condition.

**Table 2:** PLSR models constructed based on different preprocessing methods.

Type of stress	Preprocessing	Pn	Tr	gs	Ci	ΦpsII	Fv/Fm	Fv/F0	qP	NPQ	ETR
NS	R	0.377	0.331	0.354	0.424	0.334	0.376	0.231	0.254	0.350	0.445
	SG	0.416	0.355	0.361	0.483	0.354	0.424	0.335	0.374	0.472	0.525
	SG-SNV	0.399	0.359	0.37	0.493	0.356	0.423	0.35	0.399	0.494	0.546
	SG-MSC	0.393	0.351	0.365	0.518	0.348	0.420	0.344	0.398	0.492	0.545
	SG-PAN	0.411	0.352	0.39	0.536	0.339	0.410	0.340	0.395	0.469	0.547
	CWT-1	0.376	0.479	0.431	0.510	0.475	0.554	0.475	0.439	0.532	0.556
	CWT-2	0.383	0.385	0.473	0.544	0.433	0.521	0.377	0.424	0.557	0.565
	CWT-3	0.468	0.492	0.481	0.622	0.452	0.592	0.457	0.437	0.565	0.573
	CWT-4	0.476	0.493	0.488	0.676	0.495	0.595	0.471	0.447	0.636	0.639
	CWT-5	0.522	0.53	0.498	**0.702**	0.505	0.639	0.501	0.499	**0.641**	**0.655**
	CWT-6	0.503	0.511	0.473	0.698	0.516	**0.670**	0.442	0.463	0.584	0.586
	CWT-7	0.330	0.345	0.336	0.497	0.337	0.416	0.304	0.454	0.433	0.532
	CWT-8	0.373	0.355	0.285	0.434	0.321	0.368	0.288	0.374	0.302	0.438
	CWT-9	0.225	0.217	0.201	0.382	0.216	0.219	0.204	0.216	0.278	0.361
	CWT-10	0.208	0.202	0.153	0.302	0.208	0.206	0.173	0.209	0.231	0.361
SS	R	0.428	0.307	0.323	0.444	0.489	0.555	0.401	0.349	0.412	0.429
	SG	0.470	0.394	0.499	0.463	0.337	0.477	0.443	0.356	0.484	0.444
	SG-SNV	0.515	0.341	0.509	0.525	0.359	0.482	0.439	0.385	0.412	0.449
	SG-MSC	0.448	0.396	0.512	0.526	0.356	0.486	0.439	0.374	0.413	0.445
	SG-PAN	0.448	0.477	0.516	0.540	0.335	0.506	0.4391	0.373	0.423	0.436
	CWT-1	0.507	0.392	0.495	0.586	0.432	0.543	0.483	0.392	0.49	0.515
	CWT-2	0.480	0.346	0.484	0.521	0.438	0.519	0.438	0.447	0.496	0.580
	CWT-3	0.541	0.483	0.508	0.626	0.490	0.560	0.518	0.453	0.544	0.586
	CWT-4	0.513	0.489	0.545	0.639	0.497	0.573	0.528	0.51	0.589	0.603
	CWT-5	0.585	0.582	0.579	**0.665**	0.527	**0.612**	0.539	0.559	**0.629**	0.611
	CWT-6	0.591	0.558	0.565	0.613	0.59	0.519	0.506	0.524	0.613	**0.639**
	CWT-7	0.422	0.475	0.435	0.537	0.401	0.440	0.328	0.382	0.424	0.512
	CWT-8	0.450	0.490	0.478	0.464	0.345	0.457	0.322	0.377	0.369	0.461
	CWT-9	0.375	0.381	0.336	0.453	0.239	0.323	0.278	0.182	0.213	0.434
	CWT-10	0.249	0.308	0.206	0.223	0.120	0.297	0.166	0.062	0.188	0.318
NS*SS	R	0.246	0.216	0.110	0.275	0.119	0.192	0.009	0.012	0.163	0.213
	SG	0.298	0.27	0.121	0.310	0.193	0.419	0.204	0.012	0.224	0.318
	SG-SNV	0.325	0.332	0.28	0.314	0.32	0.388	0.05	0.282	0.452	0.269
	SG-MSC	0.455	0.335	0.122	0.441	0.192	0.304	0.175	0.235	0.471	0.329
	SG-PAN	0.457	0.342	0.414	0.508	0.340	0.410	0.295	0.302	0.484	0.302
	CWT-1	0.472	0.305	0.325	0.324	0.322	0.381	0.176	0.288	0.397	0.246
	CWT-2	0.474	0.378	0.359	0.543	0.302	0.412	0.324	0.045	0.454	0.348
	CWT-3	0.486	0.404	0.418	0.538	0.435	0.544	0.483	0.05	0.403	0.421
	CWT-4	0.492	0.432	0.447	**0.589**	0.481	**0.597**	0.494	0.244	0.467	**0.511**
	CWT-5	0.478	0.404	0.443	0.56	0.405	0.536	0.491	0.213	**0.524**	0.476
	CWT-6	0.486	0.343	0.382	0.488	0.432	0.506	0.474	0.221	0.435	0.367
	CWT-7	0.378	0.223	0.138	0.315	0.326	0.330	0.319	0.220	0.141	0.309
	CWT-8	0.284	0.272	0.111	0.314	0.135	0.334	0.234	0.183	0.201	0.259
	CWT-9	0.258	0.262	0.072	0.305	0.117	0.307	0.115	0.189	0.155	0.177
	CWT-10	0.209	0.221	0.101	0.299	0.013	0.170	0.099	0.088	0.171	0.020

NS, SS, and NS*SS are nitrogen stress, salt stress, and nitrogen-salt combined stress, respectively; R, Raw spectral data; SG, Savitzky-Golay; SNV, Standard normal variate; MSC, Multivariate scattering correction; PAN, Peak area normalization; CWT, Continuous wavelet transformation. The decomposition scale of CWT was set to 21 (CWT-1), 22 (CWT-2), …, 210 (CWT-10); Pn, Net photosynthetic rate; Ci, Intercellular carbon dioxide concentration; gs, Stomatal conductance; Tr, Transpiration rate; ΦPS II, Effective quantum yield of PS II photochemistry; Fv/Fm, Maximum photochemical efficiency of PS II; Fv/F0, potential activity of PS II; qP, Photochemical quenching coefficient; NPQ, Non-photochemical quenching; ETR, Electron transport rate.

Bold values representing the optimal preprocessing corresponding R2 for different indicators.

### 3.5 Spectral feature distribution

**Figure 7:**
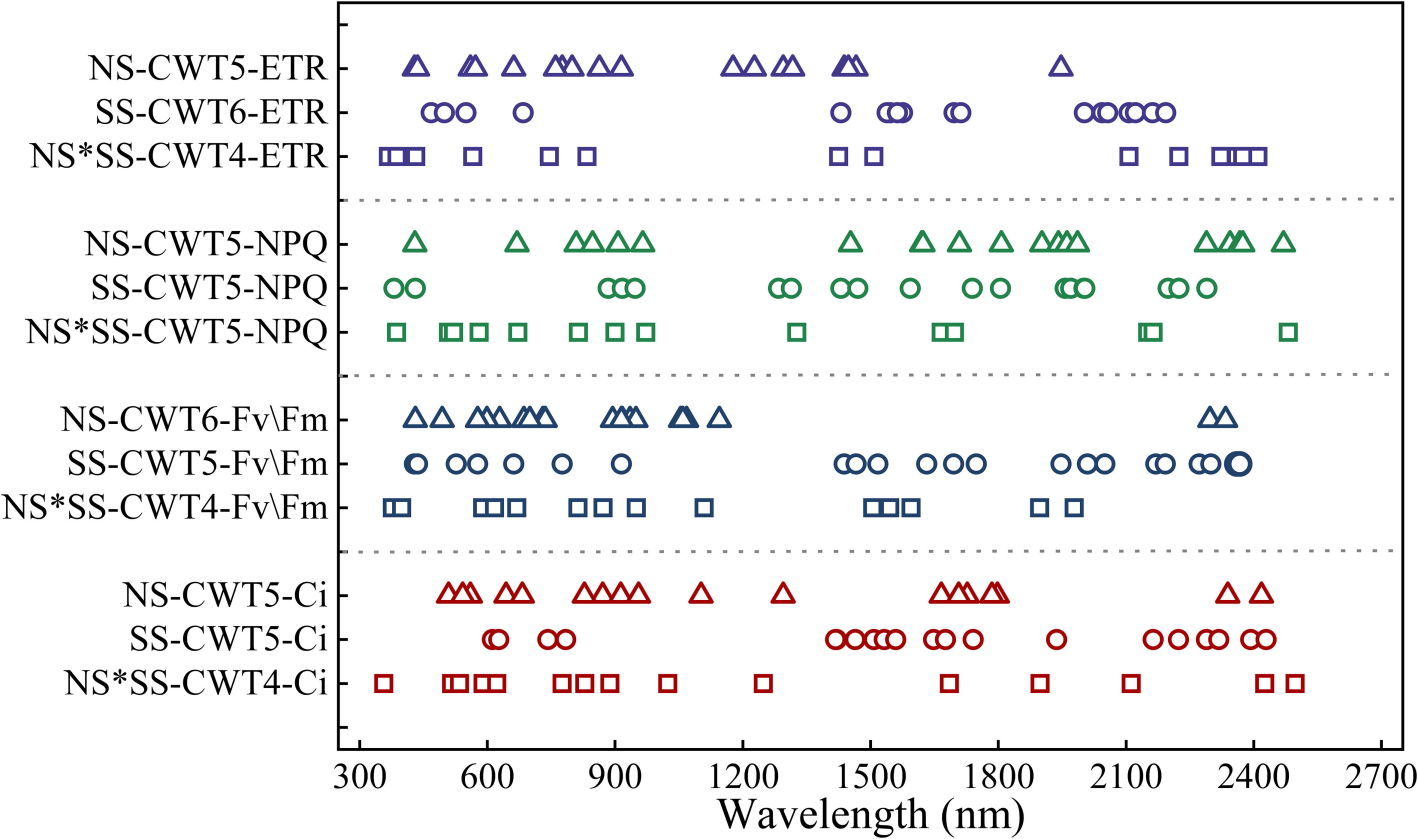
Distribution of spectral features of gas exchange and chlorophyll fluorescence parameters (PGE-CFPs) of forage rape leaves under nitrogen stress, salt stress, and nitrogen-salt combined stresses. NS, SS, and NS*SS are nitrogen stress, salt stress, and nitrogen-salt combined stresses, respectively; Ci, Intercellular carbon dioxide concentration; Fv/Fm, Maximum photochemical efficiency of PS II; NPQ, Non-photochemical quenching; ETR, Electron transport rate.

The spectral features of the parameters extracted by SPA were distributed in the red (600 – 800 nm), near-infrared, and blue-green regions. Besides, the spectral features for a parameter were different under different stresses. The Ci’s and Fv/Fm’s spectral feature distribution were similar. Specifically, most spectral features of Ci and Fv/Fm were found in the visible region under NS, visible (600 – 800 nm) and near-infrared region (1600 – 1800 and 2100 – 2500 nm) under SS, and the whole band under NS*SS. The NPQ’s spectral feature distribution were similar (800 – 1000 and 1500 – 1800 nm) under NS and SS. The ETR’s spectral features were mainly found in 500 – 900 and 1100 – 1300 nm under NS, 500 – 600, 1400 – 1700, and 2000 – 2200 nm under SS, and 400-800 and 2100 – 2500 nm under NS*SS (**Figure 7**).

### 3.6 Model construction

The SPA-extracted spectral features were used to construct PLSR, RF, and BPNN models (**Table 3**). The accuracy of the spectral features-based PLSR model was higher than that of the full band-based PLSR model (**Table 2**). The accuracy of the spectral feature-based models were different under different stresses, with the accuracy of the SSpe-based models being the highest, followed by that of NSpe- and NSpe*SSpe-based models. Besides, all constructed models generally had a higher accuracy in ETR estimation than in Ci, NPQ, and Fv/Fm estimations. By comparing the R^2^ and RMSE, the RF models had a higher accuracy than PLSR and BPNN models. The *R^2c^* for RF models for Ci estimation constructed based on NSpe, SSpe, and NSpe*SSpe were 0.814, 0.848, and 0.571, respectively, and the R^2p^ were 0.725, 0.717, and 0.369, respectively. The R^2c^ of the NSpe-, SSpe-, and NSpe*SSpe-based RF models were 0.741, 0.813 and 0.494, respectively for Fv/Fm estimation, and the R^2p^ were 0.704, 0.647, and 0.221, respectively. The R^2c^ of the RF models for NPQ estimation constructed based on NSpe, SSpe, and NSpe*SSpe were 0.781, 0.795, and 0.523, respectively, and the R^2p^ were 0.627, 0.697, and 0.273, respectively. The R^2c^ of the RF models for ETR estimation constructed based on NSpe, SSpe, and NSpe*SSpe were 0.844, 0.852, and 0.554, respectively, and the *R^2p^* were 0.707, 0.735, and 0.377, respectively.

**Table 3:** PLSR, RF, and BPNN models constructed based on the spectral features extracted by successive projections algorithm (SPA).

Parameter	Stress	PLSR				RF				BPNN			
		R^2c^	RMSE_c_	R^2p^	RMSE_p_	R^2c^	RMSE_c_	R^2p^	RMSE_p_	R^2c^	RMSE_c_	R^2p^	RMSE_p_
Ci	NS	0.585	13.101	0.541	14.054	0.814	10.908	0.717	11.705	0.819	11.121	0.56	15.547
	SS	0.773	10.498	0.644	12.471	0.848	9.738	0.725	10.432	0.824	10.841	0.701	13.451
	NS*SS	0.361	15.263	0.225	20.807	0.571	13.079	0.369	16.247	0.521	13.247	0.448	17.746
Fv/Fm	NS	0.507	0.035	0.293	0.055	0.741	0.035	0.647	0.029	0.743	0.027	0.575	0.052
	SS	0.752	0.018	0.65	0.022	0.813	0.02	0.704	0.023	0.808	0.021	0.415	0.038
	NS*SS	0.272	0.054	0.151	0.059	0.494	0.045	0.221	0.05	0.277	0.048	0.197	0.081
NPQ	NS	0.641	0.363	0.399	0.406	0.781	0.368	0.627	0.419	0.625	0.379	0.414	0.413
	SS	0.791	0.352	0.666	0.375	0.795	0.359	0.697	0.412	0.725	0.314	0.61	0.371
	NS*SS	0.352	0.389	0.154	0.446	0.523	0.411	0.273	0.442	0.429	0.401	0.388	0.454
ETR	NS	0.722	13.825	0.613	14.989	0.844	13.907	0.727	12.705	0.761	14.436	0.688	17.724
	SS	0.794	13.306	0.678	14.521	0.852	13.31	0.735	12.407	0.831	13.426	0.774	16.257
	NS*SS	0.432	16.399	0.267	20.572	0.554	17.173	0.377	15.273	0.586	16.405	0.474	19.258

NS, SS, and NS*SS are nitrogen stress, salt stress, and nitrogen-salt combined stresses, respectively; Ci, Intercellular carbon dioxide concentration; Fv/Fm, Maximum photochemical efficiency of PS II; NPQ, Non-photochemical quenching; ETR, Electron transport rate; RF, Random forest; PLSR, Partial least squares regression; BPNN, Backpropagation neural network.

### 3.7 Modeling based on feature fusion

To improve the PGE-CFPs estimation accuracy under NS*SS, the NSpe and SSpe were concatenated to construct RF model (**Table 4**). The feature fusion model had a higher accuracy than the NSpe-, SSpe-, and NSpe*SSpe-based models (**Table 2**). The R^2c^ of the spectral fusion model for Ci, Fv/Fm, NPQ, and ETR estimation were 0.878, 0.942, 0.821, and 0.893, respectively, and the R^2p^ were 0.767, 0.775, 0.714, and 0.786, respectively.

**Table 4:** RF estimation models constructed by fusing the spectral features of forage rape leaves under nitrogen stress and salt stress.

Index	R^2c^	RMSE_c_	R^2p^	RMSE_p_
N_Ci_	0.878	0.014	0.767	0.020
N_Fv/Fm_	0.942	0.008	0.775	0.011
N_NPQ_	0.821	0.001	0.714	0.020
N_ETR_	0.893	0.001	0.786	0.002

N_Ci_, Normalized Ci; N_Fv/Fm_, Normalized Fv/Fm; N_NPQ_, Normalized NPQ; N_ETR_, Normalized ETR; NS and SS are nitrogen stress and salt stress, respectively; RF, Random forest.

### 3.8 Model validation

#### 3.8.1 Pot experiment validation

**Figure 8:**
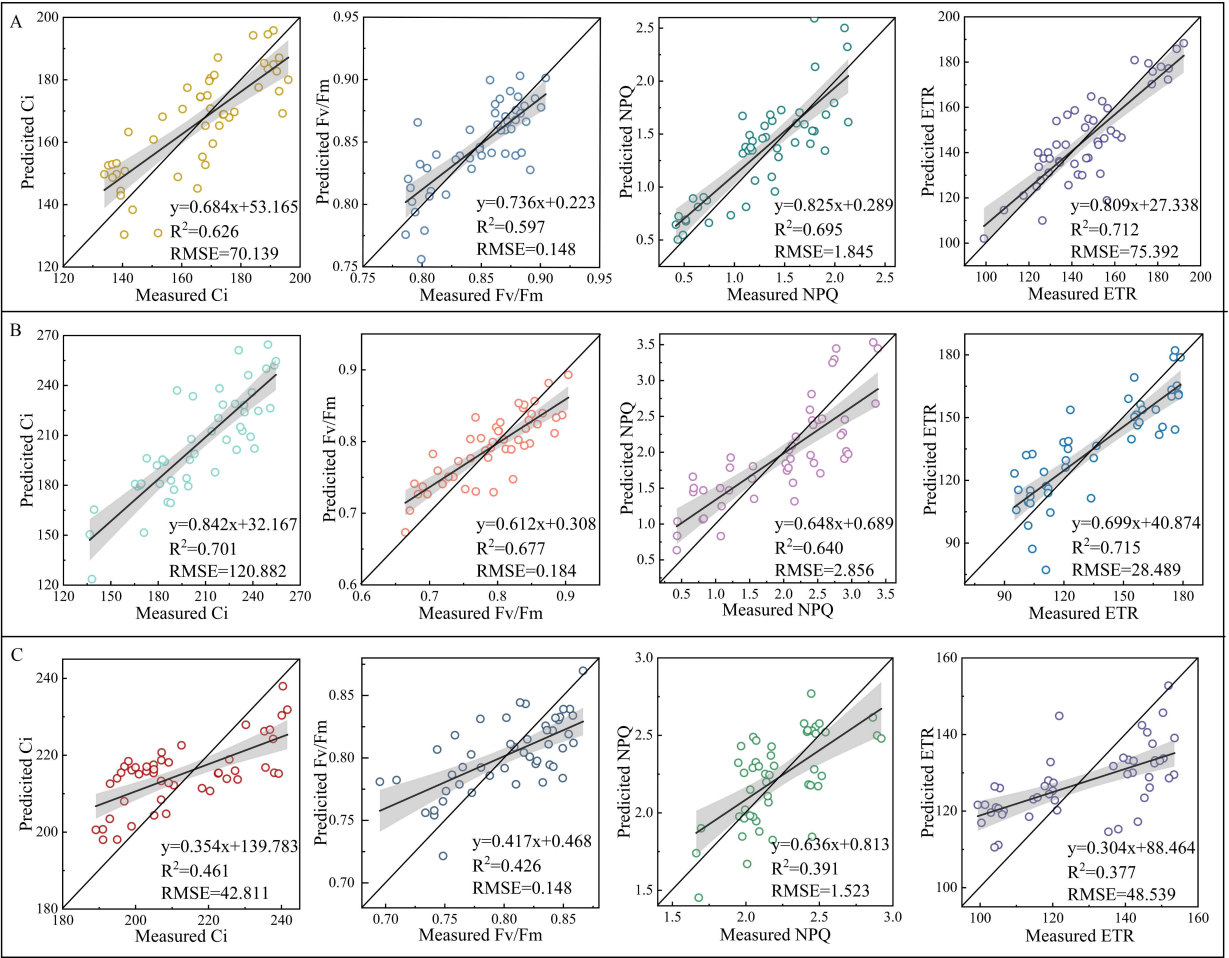
Validation of random forest (RF) models for the estimation of gas exchange and chlorophyll fluorescence parameters (PGE-CFPs) of forage rape leaves under different stresses. **(A)**, Validation of the RF models constructed using the spectral features of PGE-CFPs under nitrogen stress (NS); **(B)**, Validation of the RF models constructed using the spectral features of PGE-CFPs under salt stress (SS); **(C)**, Validation of the RF models constructed based on the fusion of spectral features under nitrogen-salt combined stresses (NS*SS). Ci, Intercellular carbon dioxide concentration; Fv/Fm, Maximum photochemical efficiency of PS II; NPQ, Non-photochemical quenching; ETR, Electron transport rate.

**Figure 9:**
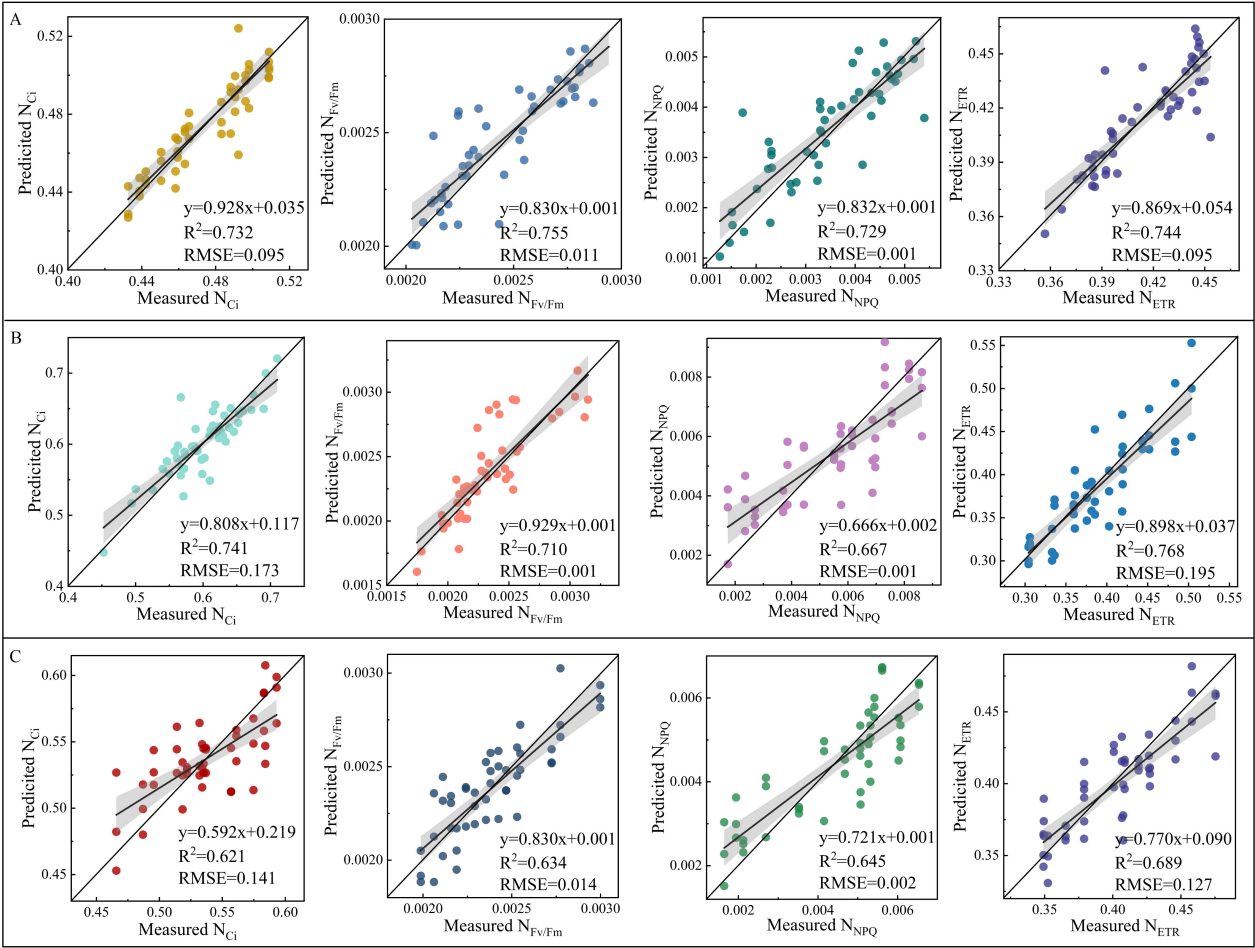
Validation of random forest (RF) model constructed based on the fusion of spectral features of gas exchange and chlorophyll fluorescence parameters (PGE-CFPs) of forage rape leaves under nitrogen stress (NS) and salt stress (SS). **(A)**, Validation of the feature fusion model for the estimation of PGE-CFPs under NS; **(B)**, Validation of the feature fusion model for the estimation of PGE-CFPs under SS; **(C)**, Validation of the feature fusion model for the estimation of PGE-CFPs under nitrogen-salt combined stresses (NS*SS). Ci, Intercellular carbon dioxide concentration; Fv/Fm, Maximum photochemical efficiency of PS II; NPQ, Non-photochemical quenching; ETR, Electron transport rate.

The validation of NSpe-, SSpe-, and NSpe*SSpe-based RF models found that the constructed models had a high accuracy. Besides, the NSpe- (R^2^: 0.712) and SS-based (R^2^: 0.715) models had a higher accuracy than the NSpe*SSpe-based model (R^2^: 0.377) (**Figure 8**). The feature fusion model had a higher accuracy than the NSpe-, SSpe-, and NSpe*SSpe-based models. The R^2^ of the feature fusion model for ETR estimation under NS and SS were 0.744 and 0.768, respectively (**Figures 9A, B**). The R^2^ of the feature fusion models for PGE-CFPs estimations under NS*SS were greater than 0.6, among which the ETR estimation accuracy was the highest, with R^2^ of 0.689 (**Figure 9C**).

#### 3.8.2 Field validation

**Figure 10:**
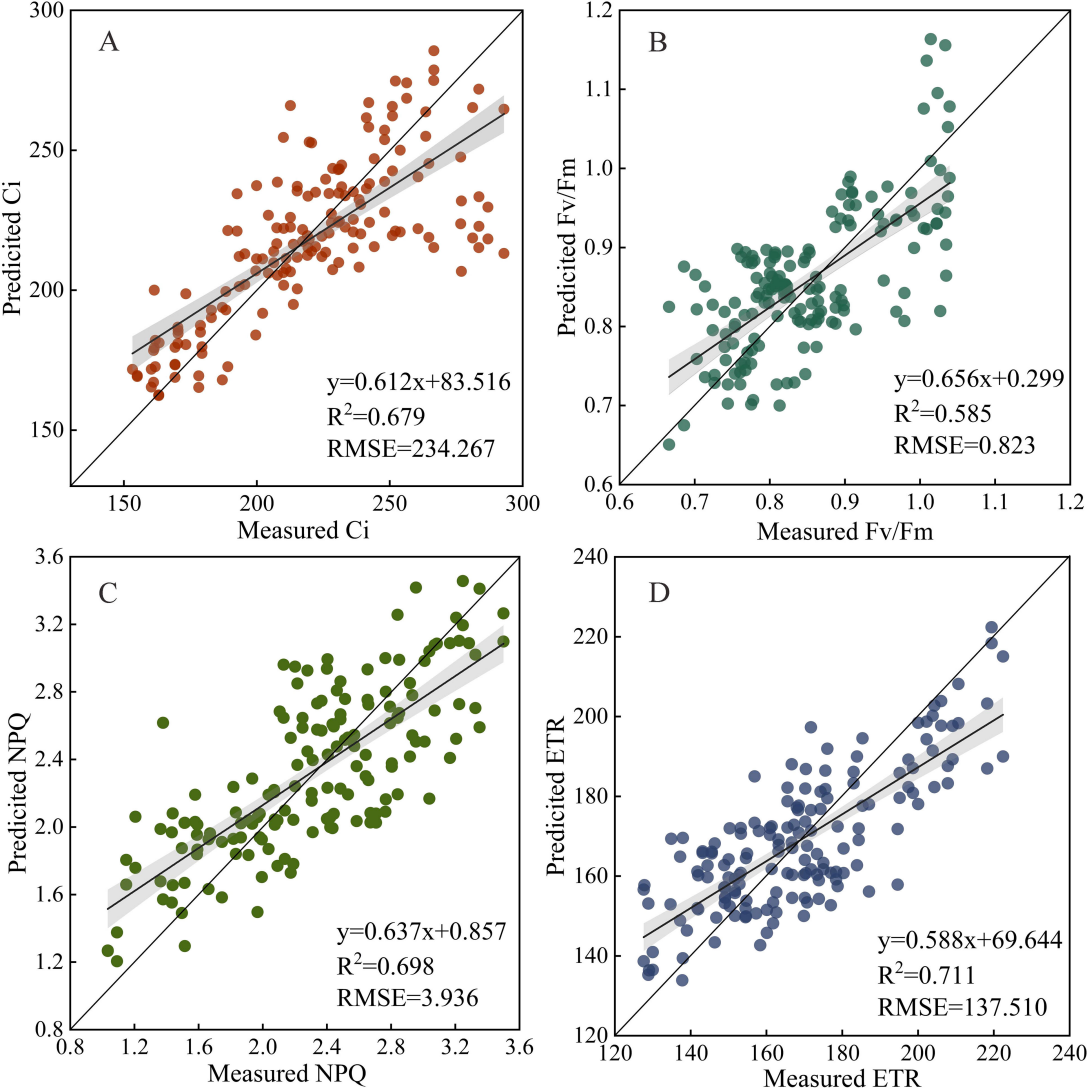
Validation of the feature fusion model using the leaf data of independent forage rape plants (n = 160). Ci, Intercellular carbon dioxide concentration; Fv/Fm, Maximum photochemical efficiency of PS II; NPQ, Non-photochemical quenching; ETR, Electron transport rate.

To further validate the transferability and practical applicability of the constructed feature fusion model, independent sample-based testing was conducted on the model using the rapeseed sample data collected from local conventional farmlands. The constructed model had high-level transferability and stability. The R^2^ of the fusion models for Ci, Fv/Fm, NPQ, and ETR estimations were 0.679, 0.585, 0.698, and 0.711, respectively, and the RMSE were 234.267, 0.823, 3.936, and 137.510, respectively (**Figure 10**).

## 4 Discussion

### 4.1 Responses of PGE-CFPs of forage rape leaves to NS, SS, and NS*SS

Crop photosynthesis is always greatly affected by external stresses, causing growth inhibition (Serbin et al., 2015). The NS and SS are currently the main abiotic stresses for crops in northwest China, and the influence mechanisms on crop photosynthetic performance are different (Rivero et al., 2014). In this research, the PGEs’ variations of forage rape leaves were consistent under NS and SS, i.e., the Pn, gs, and Tr showed a decrease trend, while the Ci showed an increase trend. However, the changes of PGEs under SS were significantly greater than that under NS. This is accordant with the study results of aquatic plants (Kumar et al., 2021) and cotton (Ibrahim et al., 2019). This may be due to the fact that (1) under SS, large amounts of Na^+^ accumulate in forage rape leaf cells, which results in Na^+^ toxicity, ion imbalance, and photosynthetic organ and chloroplast structure damages, reducing the photosynthetic rate (Farquhar and Sharkey, 1982). (2) Under NS, the concentration of pigments in chloroplasts is reduced and the stomatal limitation is increased (Taras et al., 2010), reducing photosynthetic rate. The decrease in photosynthetic rate further impacts crops’ light absorption, transformation, as well as transfer (Foyer and Noctor, 2000), in particular, it causes an obvious decrease in photochemical activities.

The photosynthetic activity of chloroplasts is easily affected by NS and SS, resulting in decreased photoenergy conversion efficiency and potential activity of PS II (Curci et al., 2017). In this research, NS and SS led to an increase in NPQ and a decrease in other fluorescence parameters. This indicates that both stresses lead to photosuppression in leaves. However, the impact mechanisms are different (Xu et al., 2018). The SS causes ion imbalance, osmotic stress, and oxidative stress, which disrupt the integrity of the thylakoid membrane, chloroplast structures, and photosynthetic electron transport chains, significantly reducing the efficiency of PS II (Munns and Tester, 2008; Siddiqui et al., 2021; Hendrickson et al., 2004). Under NS, the synthesis and functioning of cytochrome b6f, c6, and f are affected, leading to a slowdown in the electron transport chain. This reduces PS I efficiency, PS II photochemical efficiency, and finally the crop photosynthetic efficiency (Johnson et al., 2014; Shi et al., 2019; Klsch et al., 2020; Kamali et al., 2025). It should be noted that previous researches pay attention to single stresses’ impacts on crops. However, crops often face multiple stresses simultaneously, such as SS*NS. The effects of two different stresses on crops’ physiological activity are different, and the stresses jointly affect crops. This study found that compared with NS and SS, combined stresses had a greater effect on the photosynthetic performance of forage rape leaves. Under NS*SS, the insufficient supply of N reduces crops’ ability to synthesize photosynthetic pigments as well as other products; Besides, the Na^+^ toxicity and osmotic stress induced by salt stress further interfere with photosynthesis (Menezes-Benavente et al., 2004).

Studies have shown that stresses first affect crop photosynthesis, and PGE-CFPs are prioritized over other physiological indicators such as chlorophyll to signal stress (Liu et al., 2013). This study found that changes in PGE-CFPs including NPQ, Fv/Fm, ΦPS II, and ETR were large in the early stage of NS, SS, and NS*SS. This is mainly due to the fact that NPQ, Fv/Fm, ΦPS II, and ETR are important fluorescence emission parameters reflecting inhibition of PS II activity. Stress in crops leads to inhibition of PS II activity in the early stage, altering the fluorescence emission signals. This signal transduction can trigger changes in metabolic activities, hormone signaling, and gene expression in other organs, leading to diverse responses throughout the entire system (Granum et al., 2015; Dbrowski et al., 2021; Shi et al., 2019). It is important to note that crops’ PGE-CFPs contain rich photosynthesis information, and hyperspectral remote sensing can quickly detect the change of these information (Wen et al., 2022).

### 4.2 Influence of SS, NS, and SS*NS on the distribution of spectral features of PGE-CFPs of forage rape leaves

External stresses change the PGE-CFPs of crops and further leaf spectral reflectance (Dechant et al., 2017). Currently, many studies have focused on remote sensing detection of crop photosynthesis under single stresses. For example, El-Hendawy et al. (2019) used an optimized spectral index to evaluate salt-stressed wheat CFPs. Feng et al. (2015) constructed chlorophyll fluorescence index NDF12/4 for diagnosing wheat N status. However, crops are always subjected to multiple stresses simultaneously, and the response mechanism of crop leaf photosynthesis and spectra under combined stresses differs from that under single stresses (Hu et al., 2023). Buschmann et al. (2000) found that during photosynthetic electron transport in PS II, there were two fluorescence emission peaks at 690 & 740 nm. Zarco-Tejada et al. (2009) pointed out that PGE-CFPs had a close relationship with crop pigment concentration, and responded quickly to leaf biochemical properties (such as the content of proteins and amino acids) and structure variations. In the present research, the spectral features of PGE-CFPs of forage rape leaves under NS, SS, and NS*SS were extracted by SPA. It was found that the spectral features of single parameters were different under different stresses. Under NS, most spectral features of Ci and Fv/Fm were in 400 – 450 and 600 – 800 nm (visible region) (Meroni et al., 2009). The NPQ had multiple spectral features in the visible region near 987 nm, reflecting the C-H bonds in the fatty acids of forage rape leaves (Zhao et al., 2021). The spectral features of ETR were mainly distributed near 500 – 900 and 1700 nm, of which the 1725 nm band could be attributed to the stretching vibration peak of the C=O bond in macromolecules such as proteins and nucleic acids (Liang et al., 2012). Under SS, the spectral feature distribution shifted from visible region to near-infrared region, and Ci, Fv/Fm, ETR, and NPQ were found to have aggregation of spectral features at 1453, 1600, and 2250 nm (short-wave infrared region) with strong water absorption (Wang et al., 2001). Under NS*SS, spectral features were mainly distributed in 500 – 600, 1400 – 1700, and 2000 – 2200 nm. It was worth noting that under NS*SS, the spectral features extracted were less distributed in the vis region concentrated by N’s spectral features and the short wave near-infrared region concentrated by the spectral features of salt. This may be due to the fact that the cumulative effect of N and salt stresses causes significant changes in forage rape leaf components, leading to drift and other errors when extracting spectral features and non-representativeness of the selected spectral features. Of course, the specific reasons need further exploration (Berger et al., 2022; Ma et al., 2018).

### 4.3 Influence of modeling methods on PGE-CFPs estimation under combined stresses

**Figure 11:**
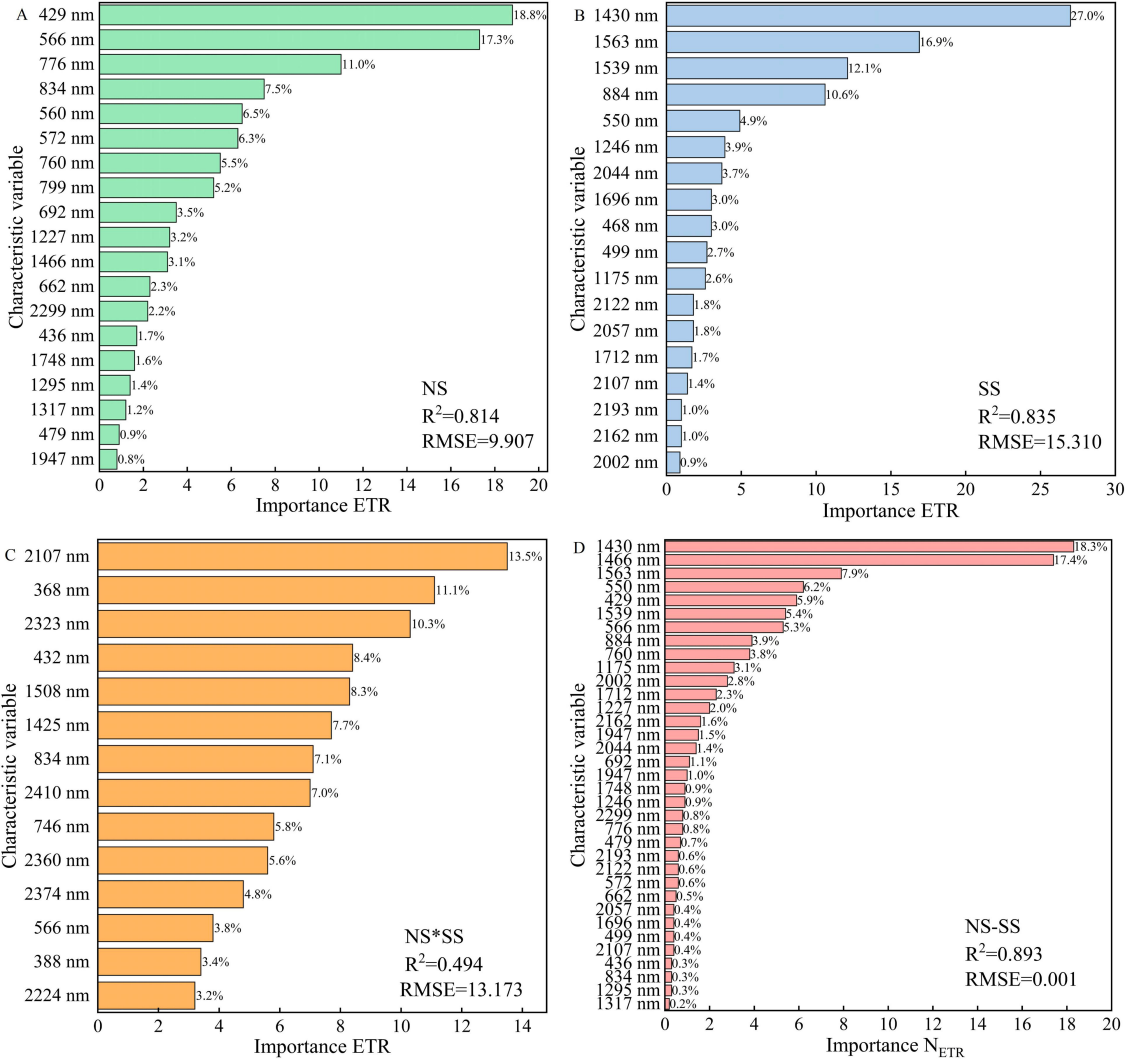
The importance of spectral features in the ETR estimation based on random forest regression under different stresses. **(A)**, The model constructed based on the spectral features of gas exchange and chlorophyll fluorescence parameters (PGE-CFPs) of forage rape leaves under nitrogen stress (NS); **(B)**, The model constructed based on the spectral features of PGE-CFPs under salt stress (SS); **(C)**, The model constructed based on the spectral features of PGE-CFPs under nitrogen-salt combined stresses (NS*SS); **(D)**, The model constructed based on the fusion of spectral features of PGE-CFPs under NS and SS (NS-SS). ETR, Electron transport rate.

Modeling variables and methods significantly affect the accuracy of spectral estimation (El-Hendawy et al., 2019). In the present research, PLSR, RF, and BPNN modeling were completed using SPA-extracted features. It was found that RF model had a higher accuracy than the other two. The reason is that RF regression can better deal with the bias in data that has a large impact on the estimation results, and the randomness of sampling and feature selection makes the model less prone to overfitting (Breiman, 2001). It was worth noting that in this research, the RF model had a high accuracy in estimating PGE-CFPs of forage rape leaves under NS and SS, among which the accuracy in estimating ETR was the highest (R^2^: 0.844 and 0.852, respectively). However, the R^2^ was only 0.554 in ETR estimation under NS*SS. To find out the reason, the importance of the features participating in the RF modeling under different stresses was evaluated. Under NS, pigments’ spectral features in the visible region contributed significantly to the model accuracy (**Figure 11A**) (Meroni et al., 2009). Under SS, the spectral features of short wave near-infrared region contributed greatly to the model accuracy (**Figure 11B**) (Wang et al., 2001). Besides, under NS*SS, the spectral features with significant contributions to the model accuracy were at 2107, 368, and 2323 nm, which were obviously different from those under SS and NS (**Figure 11C**). This may explain the low accuracy of the estimation model under NS*SS.

Under multiple stresses, the cumulative effect of the stresses often occurs (Cotrozzi and Couture, 2020). The NS inhibits crop photosynthesis, and the SS reduces crop absorption of nutrients and water by damaging photosynthetic and defense systems. However, due to the variability of environmental conditions and the heterogeneity caused by some duplicate samples, the estimation accuracy is not ideal (Couture et al., 2016). To increase the accuracy of forage rape photosynthetic performance estimation under SS*NS, a new modeling strategy, fusing the spectral features of PGE-CFPs of forage rape leaves under SS and NS was proposed. It was found that the feature fusion model had a higher accuracy than the SSpe-, NSpe-, and SSpe*NSpe-based models, and the contribution to the model accuracy of SSpe was greater than that of NSpe. This indicates that in the feature fusion model, the SS plays a more important role than the NS (**Figure 11D**). Besides, the validation of the feature fusion model in estimating PGE-CFPs of forage rape leaves under SS, NS, and NS*SS (**Figure 9**) showed that feature fusion significantly improved the PGE-CFPs estimation accuracy under SS*NS, and the accuracy of ETR estimation was the maximum (R^2^: 0.689). This may be due to the fact that feature fusion modeling strategy only considers the influence of SS and NS on the PGE-CFPs of forage rape leaves under NS*SS, and avoids other environmental factors’ influences and the two stresses’ cumulative effect. This can significantly limit other factors’ disturbance, increasing the estimation accuracy (Cotrozzi and Couture, 2020). In general, this study confirms that in the early stage of stresses, photosynthesis preferentially exhibits stress symptoms, and forage rape growth status can be evaluated by hyperspectral remote sensing combined with feature fusion. However, the obtained study results are based solely on data from forage rape. The applicability of this model to other crops such as corn and rice is not clear. Therefore, further verification research will be conducted on different crops in the future.

## 5 Conclusion

This study clarified the distribution of the SPA-extracted spectral features of PGE-CFPs of forage rape leaves under NS, SS, and NS*SS based on CWT preprocessing, and constructed the optimal PGE-CFPs estimation model based on random forest. The proposed method improved the PGE-CFPs estimation accuracy under NS*SS. Different spectral preprocessing methods (SG smoothing, SG-SNV transform, SG-MSC, SG-PAN, and SG-CWT) combined with SPA could effectively extract PGE-CFPs’ spectral features of forage rape leaves under different stresses, and RF model had a higher accuracy than the PLSR and BPNN models among the NSpe-, SSpe-, and NSpe*SSpe-based models. However, the RF model showed a low accuracy in estimating PGE-CFPs under NS*SS. Therefore, this study constructed the estimation model by fusing PGE-CFPs’ spectral features of forage rape leaves under NS and SS, which obviously increased the accuracy of photosynthesis performance estimation under NS*SS (R^2^: 0.621 – 0.689). Especially, the feature fusion model had a highest accuracy in ETR estimation (R^2^: 0.689). This research results will offer a reference for improving the accuracy of photosynthetic performance estimation under salt-nitrogen combined stresses.

## Data Availability

The raw data supporting the conclusions of this article will be made available by the authors, without undue reservation.

## References

[B1] Baker N. R. Rosenqvist E. (2004). Applications of chlorophyll fluorescence can improve crop production strategies: An examination of future possibilities. J. Exp. Bot. 55, 1607–1621. doi: 10.1093/jxb/erz535 15258166

[B2] Barnes R. Dhanoa M. Lister S. (1993). Letter: Correction to the description of standard normal variate (SNV) and de-trend (DT) ransformations in practical spectroscopy with applications in food and everage analysis-2nd edition. J. Near Infrared Spectrosc. 1, 185–186. doi: 10.1255/jnirs.21

[B3] Berger K. Machwitz M. Kycko M. Kefauver S. C. Van Wittenberghe S. Gerhards M.. (2022). Multi-sensor spectral synergies for crop stress detection and monitoring in the optical domain: a review. Remote Sens. environ. 280, 113198. doi: 10.1016/j.rse.2022.113198 36090616 PMC7613382

[B4] Breiman I. (2001). Random forest. Mach. learn. 45, 5–32. doi: 10.1023/A:1010933404324

[B5] Buschmann C. Langsdorf G. Lichtenthaler H. K. (2000). Imaging of the blue, green, and red fluorescence emission of plants: an overview. Photosynthetica 38, 483–491. doi: 10.1023/A:1012440903014

[B6] Chen J. Y. Yao X. Huang F. Liu Y. Yu Q. Wang N.. (2016). N status monitoring model in winter wheat based on image processing. Trans. Chin. Soc. Agric. Eng. 32, 163–170. doi: 10.11975/j.issn.1002-6819.2016.04.023

[B7] Clarke K. R. Green R. H. (1988). Statistical design and analysis for a ‘biological effects’ study. Mar. Biol. 46, 213–226. doi: 10.3354/meps046213

[B8] Cotrozzi L. Couture J. J. (2020). Hyperspectral assessment of plant responses to multi-stress environments: Prospects for managing protected agrosystems. Plants People Planet. 2, 244–258. doi: 10.1002/ppp3.10080

[B9] Cotty T. L. Dorin B. (2012). A global foresight on food crop needs for livestock. Animal 6, 1528–1536. doi: 10.1017/S1751731112000377 23031526

[B10] Couture J. J. Singh A. Rubert-Nason K. F. Serbin S. P. Lindroth R. L. Townsend P. A. (2016). Spectroscopic determination of ecologically relevant plant secondary metabolites. Methods Ecol. Evol. 7, 1402–1412. doi: 10.1111/2041-210X.12596

[B11] Curci P. L. Cigliano R. A. Zuluaga D. L. Janni M. Sanseverino W. Sonnante G. (2017). Transcriptomic response of durum wheat to nitrogen starvation. Sci. Rep. 7, 1–14. doi: 10.1038/s41598-017-01377-0 28446759 PMC5430780

[B12] Dbrowski P. Baczewska-Dbrowska A. H. Bussotti F. Pollastrini M. Piekut K. Kowalik W.. (2021). Photosynthetic efficiency of microcystis ssp. under salt stress. Environ. Exp. Bot. 186, 104459. doi: 10.1016/j.envexpbot.2021.104459

[B13] Dechant B. Cuntz M. Vohland M. Schulz E. Doktor D. (2017). Estimation of photosynthesis traits from leaf reflectance spectra: Correlation to nitrogen content as the dominant mechanism. Remote Sens. Environ. 196, 279–292. doi: 10.1016/j.rse.2017.05.019

[B14] El-Hendawy S. E. Al-Suhaibani N. A. Hassan W. M. Dewir Y. H. Elsayed S. Al-Ashkar I.. (2019). Evaluation of wavelengths and spectral reflectance indices for high-throughput assessment of growth, water relations and ion contents of wheat irrigated with saline water. Agric. Water Manage. 212, 358–377. doi: 10.1016/j.agwat.2018.09.009

[B15] Farquhar G. D. Sharkey T. D. (1982). Stomatal conductance and photosynthesis. Annu. Rev. Plant Physiol. 33, 317–345. doi: 10.1146/annurev.pp.33.060182.001533

[B16] Feng W. He L. Zhang H. Y. Guo B. B. Zhu Y. J. Wang C. Y.. (2015). Assessment of plant nitrogen status using chlorophyll fluorescence parameters of the upper leaves in winter wheat. Eur. J. Agro. 64, 78–87. doi: 10.1016/j.eja.2014.12.013

[B17] Foyer C. H. Noctor G. (2000). Tansley review No. 112. Oxygen processing in photosynthesis: Regulation and signalling. New Phytol. 146, 359–388. doi: 10.1046/j.1469-8137.2000.00667.x

[B18] Galvo R. K. H. Araújo M. C. U. Fragoso W. D. Silva E. C. Paiva H. M. (2007). A variable elimination method to improve the parsimony of MLR models using the successive projections algorithm. Chemo. Intell. Lab. Sys. 92, 83–91. doi: 10.1016/j.chemolab.2007.12.004

[B19] Granum E. Pérez-Bueno M. L. Barón M. (2015). Metabolic responses of avocado plants to stress induced by Rosellinia necatrix analysed by fluorescence and thermal imaging. Eur. J. Plant Pathol. 142, 625–632. doi: 10.1007/s10658-015-0640-9

[B20] Hendrickson L. Furbank R. T. Chow W. S. (2004). A simple alternative approach to assessing the fate of absorbed light energy using chlorophyll fluorescence. Photosynth. Res. 82, 73–81. doi: 10.1023/B:PRES.0000040446.87305.f4 16228614

[B21] Hu Y. Yang F. Yang N. Jia W. Cui Y. (2023). Analysis and prospects of saline-alkali land in China from the perspective of utilization. Chin. J. Soil Sci. 54, 489–494. doi: 10.19336/j.cnki.trtb.2022031902

[B22] Huang Z. A. Jiang D. A. Yang Y. Sun J. W. Jin S. H. (2004). Effects of nitrogen deficiency on gas exchange, chlorophyll fluorescence, and antioxidant enzymes in leaves of rice plants. Photosynthetica 42, 357–364. doi: 10.1023/B:PHOT.0000046153.08935.4c

[B23] Ibrahim W. Qiu C. Zhang C. Cao F. Shuijin Z. Wu F. (2019). Comparative physiological analysis in the tolerance to salt and drought individual and combination in two cotton genotypes with contrasting salt tolerance. Physiol. Plant. 165, 155–168. doi: 10.1111/ppl.12791 30006979

[B24] Inoue Y. Guérif M. Baret F. Skidmore A. Gitelson A. Schlerf M.. (2016). Simple and robust methods for remote sensing of canopy chlorophyll content: A comparative analysis of hyperspectral data for different types of vegetation. Plant Cell Environ. 39, 2609–2623. doi: 10.1111/pce.12815 27650474

[B25] Johnson M. P. Vasilev C. Olsen J. D. Hunter C. N. (2014). Nanodomains of cytochrome b(6)f and photosystem ii complexes in spinach grana thylakoid membranes. Plant Cell. 26, 3051–3061. doi: 10.1105/tpc.114.127233 25035407 PMC4145131

[B26] Kamali S. Sonkar K. Singh A. (2025). Cellular transport and multifaceted roles of jasmonates in nutrient deficiency response in plants. J. Plant Growth Regul. 44, 115–130. doi: 10.1007/s00344-024-11364-1

[B27] Klsch A. Radon C. Golub M. Baumert A. Mielke T. Lisdat F.. (2020). Current limits of structural biology: the transient interaction between cytochrome c and photosystem i. Curr. Res. Struct. Biol. 2, 171–179. doi: 10.1016/j.crstbi.2020.08.003 34235477 PMC8244401

[B28] Koger C. H. Bruce L. M. Shaw D. R. Reddy K. N. (2003). Wavelet analysis of hyperspectral reflectance data for detecting pitted morningglory (Ipomoea lacunosa) in soybean (Glycine max). Remote Sens. Environ. 86, 108–119. doi: 10.1016/S0034-4257(03)00071-3

[B29] Konapala G. Mishra A. K. Wada Y. Mann M. E. (2020). Climate change will affect global water availability through compounding changes in seasonal precipitation and evaporation. Nat. Commun. 11, 3044. doi: 10.1038/s41467-020-16757-w 32576822 PMC7311549

[B30] Kumar S. Li G. Yang J. Huang X. Ji Q. Liu Z.. (2021). Effect of salt stress on growth, physiological parameters, and ionic concentration of water dropwort (Oenanthe javanica) cultivars. Front. Plant Sci. 12. doi: 10.3389/fpls.2021.660409 PMC8256277 34234795

[B31] Li B. (2021). Development of PS II potential maximum photosynthetic capacity detector for protected crops based on visible-near infrared technology (Northwest A & F University). doi: 10.27409/d.cnki.gxbnu.2021.001576

[B32] Li R. C. Lin H. L. (2014). Developing the agro-grassland system to insure food security of China. J. Agric. Chem. Environ. 3, 9–15. doi: 10.4236/jacen.2014.33B002

[B33] Liang H. Q. Tao Y. P. Han L. G. Yun X. Yu J. (2012). Raman, FTIR spectra and normal mode analysis of acetanilide. Spectrosc. Spectral Anal. 32, 2706–2709. doi: 10.3964/j.issn.1000-0593(2012)10-2706-04 23285870

[B34] Liu L. Y. Zhang Y. J. Jiao Q. J. Peng D. L. (2013). Assessing photosynthetic light-use efficiency using a solar-induced chlorophyll fluorescence and photochemical reflectance index. Int. J. Remote Sensing. 34, 4264–4280. doi: 10.1080/01431161.2013.775533

[B35] Liu X. Zhang R. Yang X. Yu T. Cao W. X. Hua L. M.. (2024). Study on soil salt content, degree and type of salinization in Minqin Basin. Grassland Turf. 44, 125–134. doi: 10.13817/j.cnki.cyycp.2024.02.013

[B36] Luo J. X. (1985). Improvement of Saline Alkaline Land in Xinjiang Reclamation Area (Beijing: Water Resources and Electric Power Press).

[B37] Ma B. D. Pu R. L. Zhang S. Wu L. X. (2018). Spectral identification of stress types for maize seedlings under single and combined stresses. IEEE Access. 6, 13773–13782. doi: 10.1109/ACCESS.2018.2810084

[B38] Menezes-Benavente L. Kernodle S. P. Margis-Pinheiro M. Scandalios J. G. (2004). Salt-induced antioxidant metabolism defenses in maize (Zea mays L.) seedlings. Redox report: Commun. Free Radical Res. 9, 29–36. doi: 10.1179/135100004225003888 15035825

[B39] Meroni M. Rossini M. Guanter L. Alonso L. Rascher U. Colombo R.. (2009). Remote sensing of solar-induced chlorophyll fluorescence: review of methods and applications. Remote Sens. Environ. 113, 2037–2051. doi: 10.1016/j.rse.2009.05.003

[B40] Munns R. Tester M. (2008). Mechanisms of salt tolerance. Annu. Rev. Plant Biol. 59, 651–681. doi: 10.1146/annurev.arplant.59.032607.092911 18444910

[B41] Okyere F. G. Cudjoe D. K. Virlet N. Castle M. Riche A. B. Greche L.. (2024). Hyperspectral imaging for phenotyping plant drought stress and nitrogen interactions using multivariate modeling and machine learning techniques in wheat. Remote Sensing. 16, 3446. doi: 10.3390/rs16183446

[B42] Peñuelas J. Isla R. Filella I. Araus J. L. (1997). Visible and near-infrared reflectance assessment of salt effects on barley. Crop Sci. 37, 198–202. doi: 10.2135/cropsci1997.0011183X003700010033x

[B43] Porcar-Castell A. Tyystjärvi E. Atherton J. van der Tol C. Flexas J. Pfündel E. E.. (2014). Linking chlo-rophyll a fluorescence to photosynthesis for remote sensing applications: mechanisms and challenges. J. Exp. Bot. 65, 4065–4095. doi: 10.1093/jxb/eru191 24868038

[B44] Rivero R. M. Rosa M. Shulaev V. Blumwald E. Suzuki N. (2014). Abiotic and biotic stress combinations. New Phytol. 203, 32–43. doi: 10.1111/nph.12797 24720847

[B45] Serbin S. P. Singh A. Desai A. R. Dubois S. G. Jablosnki A. D. Kingdon C. C.. (2015). Remotely estimating photosynthetic capacity, and its response to temperature, in vegetation canopies using imaging spectroscopy. Remote Sens. Environ. 167, 78–87. doi: 10.1016/j.rse.2015.05.024

[B46] Shi L. R. Bai L. R. Guo X. L. (2019). The effect of low nitrogen stress on the growth and rapid chlorophyll fluorescence kinetic parameters of different varieties of triticale seedlings. Chin. J. Grassland. 41, 37–42. doi: 10.16742/j.zgcdxb.20190037

[B47] Siddiqui S. A. Khatri K. Patel D. Rathore M. S. (2021). Photosynthetic gas exchange and chlorophyll a fluorescence in salicornia brachiata (roxb.) under osmotic stress. J. Plant Growth Regul. 41, 1–16. doi: 10.1007/s00344-021-10311-8

[B48] Tan C. W. Huang W. J. Jin X. L. Wang J. C. Tong L. Wang J. H.. (2012). Using hyperspectral vegetation index to monitor the chlorophyll fluorescence parameters Fv/Fm of compact corn. Spectrosc. Spectral Anal. 32, 1287–1291. doi: 10.3964/j.issn.1000-0593(2012)05-1287-05 22827074

[B49] Taras A. Heta M. Marja H. Y. Taina T. Esa T. (2010). Acclimation of photosynthesis to nitrogen deficiency in Phaseolus vulgaris. Planta 232, 887–898. doi: 10.1007/s00425-010-1227-5 20632184

[B50] Tirado S. B. Dennis S. S. Enders T. A. Springer N. M. (2020). Utilizing top-down hyperspectral imaging for monitoring genotype and growth conditions in maize. Cold Spring Harbor Lab. 23. doi: 10.1101/2020.01.21.914069

[B51] Wang J. G. Tian T. Wang H. J. Cui J. Shi X. Y. Song J. H.. (2023). Improving the estimation accuracy of rapeseed leaf photosynthetic characteristics under salinity stress using continuous wavelet transform and successive projections algorithm. Front. Plant Sci. 14. doi: 10.3389/fpls.2023.1284172 PMC10733793 38130483

[B52] Wang J. H. Zhao C. J. Guo X. W. Tian Q. J. (2001). Study on the water status of wheat leaves diagnosed by the spectral reflectance. Sci. Agric. Sinica. 1, 104–107.

[B53] Wang L. Zeng Y. Chen T. (2015). Back propagation neural network with adaptive differential evolution algorithm for time series forecasting. Expert Systems with Applications. 42, 855–863. doi: 10.1016/j.eswa.2014.08.018

[B54] Wen S. Y. Shi N. Lu J. W. Gao Q. W. Hu W. R. Cao Z. D. Y.. (2022). Continuous wavelet transform and back propagation neural network for condition monitoring chlorophyll fluorescence parameters Fv/Fm of rice leaves. Agriculture 12, 1197–1197. doi: 10.3390/AGRICULTURE12081197

[B55] Xing L. Goldsmith P. (2013). Improving Chinese soybean meal demand estimation by addressing the non-commercial. China Agric. Econ. Review. 5, 543–566. doi: 10.1108/caer-06-2012-0069

[B56] Xu Y. L. Wu L. Wu W. J. Ma Y. H. Xu H. J. Fan S. (2018). Effects of nitrogen application on wheat yield and runoff loss of nitrogen and application threshold of nitrogen fertilizer. J. Soil Water Conserv. 32, 246–251. doi: 10.13870/j.cnki.stbcxb.2018.02.036

[B57] Xue H. Y. Zhang Y. J. Liu L. T. Sun H. C. Li C. D. (2013). Responses of spectral reflectance, photosynthesis and chlorophyll fluorescence in cotton during drought stress and rewatering. Scientia Agricult. Sinica. 46, 2386–2393. doi: 10.3864/j.issn.0578-1752.2013.11.024

[B58] Zahra J. Nazim H. S. Cai G. Han Y. Zhang G. P. (2014). The influence of salt on cell ultrastructures and photosynthetic apparatus of barley genotypes differing in salt stress tolerance. Acta Physiol. Plant. 36, 1261–1269. doi: 10.1007/s11738-014-1506-z

[B59] Zandalinas S. I. Mittler R. (2022). Plant responses to multifactorial stress combination. New Phytol. 234, 1161–1167. doi: 10.1111/NPH.18087 35278228

[B60] Zarco-Tejada P. J. Berni J. A. J. Suárez L. Sepulcre-Cantó G. Morales F. Miller J. R. (2009). Imaging chlorophyll fluorescence with an airborne narrow-band multispectral camera for vegetation stress detection. Remote Sens. Environ. 113, 1262–1275. doi: 10.1016/j.rse.2009.02.016

[B61] Zarco-Tejada P. J. Miller J. R. Mohammed G. H. Noland T. L. Sampson P. H. (2000). Chlorophyll fluorescence effects on vegetation apparent reflectance: II. Laboratory and airborne canopy-level measurements with hyperspectral data. Remote Sens. Environ. 74, 596–608. doi: 10.1016/S0034-4257(00)00149-8

[B62] Zeng G. P. Tao S. (2023). A generalized linear transformation and its effects on logistic regression. Mathematics 11, 467–467. doi: 10.3390/MATH11020467

[B63] Zhang H. Hu H. Zhang X. B. Wang K. L. Song T. Q. Zeng F. P. (2012). Detecting Suaeda salsa L. chlorophyll fluorescence response to salt stress by using hyperspectral reflectance. Acta Physiol. Plant. 34, 581–588. doi: 10.1007/s11738-011-0857-y

[B64] Zhao R. M. An L. L. Song D. Li M. Z. Qiao L. Sun H. (2021). Detection of chlorophyll fluorescence parameters of potato leaves based on continuous wavelet transform and spectral analysis. Spectro. Acta Part A: Mol. Biomol. Spectrosc. 259, 119768. doi: 10.1016/J.SAA.2021.119768 33971438

[B65] Zheng W. Lu X. Li Y. Li S. Zhang Y. Z. (2021). Hyperspectral identification of chlorophyll fluorescence parameters of suaeda salsa in coastal wetlands. Remote Sensing. 13, 2066. doi: 10.3390/rs13112066

[B66] Zhu Q. Q. Liu G. H. Xu Y. M. Yang J. Y. Zhang Y. H. (2019). Effect of water and nitrogen on the yield and quality of forage rape grown after wheat in South Xinjiang. Chin. J. Eco-Agricult. 27, 1033–1041. doi: 10.13930/j.cnki.cjea.180997

[B67] Zhu Y. Tian Y. C. Ma J. F. Yao X. Liu X. J. Cao W. X. (2007). Relationship between chlorophyll fluorescence parameters and reflectance spectrum characteristics of wheat leaves. Acta Agro. Sinica. 33, 1286–1292.

